# Roles of Oxidative Stress and Nrf2 Signaling in Pathogenic and Non-Pathogenic Cells: A Possible General Mechanism of Resistance to Therapy

**DOI:** 10.3390/antiox12071371

**Published:** 2023-06-30

**Authors:** Mira Hammad, Mohammad Raftari, Rute Cesário, Rima Salma, Paulo Godoy, S. Noushin Emami, Siamak Haghdoost

**Affiliations:** 1 University of Caen Normandy, UMR6252 CIMAP/ARIA, GANIL, 14000 Caen, France; 2 Department of Molecular Biosciences, The Wenner-Gren Institute, Stockholm University, 10691 Stockholm, Sweden; 3 Natural Resources Institute, University of Greenwich, London ME4 4TB, UK; 4 Advanced Resource Center for HADrontherapy in Europe (ARCHADE), 14000 Caen, France

**Keywords:** oxidative stress, ROS: Nrf2 signaling, stem cell differentiation, adipogenesis, osteogenesis, infection diseases, malaria, cancer, cancer stem cell, treatment resistance

## Abstract

The coordinating role of nuclear factor erythroid-2-related factor 2 (Nrf2) in cellular function is undeniable. Evidence indicates that this transcription factor exerts massive regulatory functions in multiple signaling pathways concerning redox homeostasis and xenobiotics, macromolecules, and iron metabolism. Being the master regulator of antioxidant system, Nrf2 controls cellular fate, influencing cell proliferation, differentiation, apoptosis, resistance to therapy, and senescence processes, as well as infection disease success. Because Nrf2 is the key coordinator of cell defence mechanisms, dysregulation of its signaling has been associated with carcinogenic phenomena and infectious and age-related diseases. Deregulation of this cytoprotective system may also interfere with immune response. Oxidative burst, one of the main microbicidal mechanisms, could be impaired during the initial phagocytosis of pathogens, which could lead to the successful establishment of infection and promote susceptibility to infectious diseases. There is still a knowledge gap to fill regarding the molecular mechanisms by which Nrf2 orchestrates such complex networks involving multiple pathways. This review describes the role of Nrf2 in non-pathogenic and pathogenic cells.

## 1. Introduction

Free radicals are species that contain one single electron at an atomic or molecular valence orbital. These species are formed by the gain or loss of an electron, comprising anion and cation radicals specifically, or by homolytic fission, which occurs when a covalent bond is broken in a way where each electron remains in each of the two molecules/atoms previously bonded [1]. Indeed, organic covalent bonds like C-C, C-H, or C-O are extremely strong, requiring a high energy for bond dissociation, which can be obtained by the absorption of ultraviolet (UVR) or ionizing radiation (IR), for example. However, exposure to biological tissue by ionising radiation also results in water radiolysis, which constitutes the main source of reactive oxygen species (ROS). Reactive nitrogen species (RNS), reactive sulfur species (RSS), and carbon-centered radicals (R^•^) are also common within the intracellular environment. Overall, free radicals are produced as by-products of cellular metabolism and aerobic respiration. In addition, since free radicals are vital for proper redox signaling, cells are also equipped with enzymatic systems that deliberately produce ROS, such as NADPH oxidase [2]. Apart from endogenous sources, several external agents, e.g., drugs, food, toxins, ionizing radiation, and pollutants, can increase the physiological levels of free radicals [3]. Free radicals aim to stabilize their unpaired electrons. The vast majority pair it with an electron extracted from an oxidised molecule or donate it to nearby biomolecules, thus reducing them. Under these redox reactions, unstable free radicals can interact with all classes of macromolecules such as proteins, lipids, carbohydrates, and nucleic acids [4] and modify their structures. Additionally, free radicals can also be stabilized by undergoing addition, disproportionation, or self-annihilation reactions [5,6]. 

ROS have varying effects, including the induction of oxidative damage to biomolecules and signal activation, depending on their intracellular levels, which are tightly regulated by the level of available enzymatic and non-enzymatic antioxidants. Nrf2, as a key regulator, controls the expression of several enzymatic antioxidants and results in the modulation of the levels of ROS, such as superoxide dismutase (SOD), catalase (CAT), glutathione peroxidase (GPx), and heme oxygenase-1 (HO-1), which are vital in maintaining redox balance and cellular homeostasis.

Cellular response to ROS includes reversible redox signaling or irreversible non-enzymatic reactions, which depend on the type and concentration of the ROS. ROS include both radical species such as hydroxyl radical and hydroxyl radicals and non-radical species such as hydrogen peroxide, peroxynitrite, and hypochlorous acid. Radical species are mainly responsible for the induction of oxidative damage to biomolecules, while non-radical species are mainly involved in the signaling and second messenger functions. Non-radical species have pronounced effects on proteins, particularly on the cysteine residue of a protein. Cysteine residues can be oxidized by hydroperoxides and other reactive molecules such as thiyl radicals and sulfenic acids and can result in the changing of the catalytic activities of proteins/enzymes, effecting the redox regulation of protein function or signaling pathways [7]. Notably, compared with the effects of post-translational modifications such as phosphorylation, the effects of the oxidation of cysteine on the function of and changes in protein structures are less characterized. ROS engage in multiple cell pathways as secondary messengers by promoting, e.g., cysteine oxidation in proteins, leading to the modification of protein structures, which may result in aggregation or degradation with involvement in some pathological conditions [8]. Under physiological conditions, known as oxidative eustress, redox signaling contributes to cellular homeostasis, influencing cellular growth rate, stemness/differentiation/development states [9,10], immune function [11,12], and adaptive responses [13,14]. 

On the other hand, excessive levels of ROS lead to the nonspecific oxidation of biomolecules, which culminates in reversible or irreversible damage to macromolecules and the disruption of redox signaling. This toxic cellular state, referred to as oxidative distress, compromises cellular homeostasis as it promotes genomic instability, protein denaturation and aggregation, membrane abnormalities, organelle dysfunction, etc. [15]. These negative repercussions can delay the cell cycle and promote cell senescence [16] and death [17].

To avoid these harmful conditions, cells and organisms activate adaptive responses, for example during physical activity, that neutralize ROS or delay their subsequent reactions [18]. Many of these detoxifying agents are capable of directly stabilizing free radicals or reverting them to some extent oxidative injuries, these being collectively referred to as antioxidants. These include endogenous and exogenous scavengers with and without enzymatic activity. Thus, oxidative eustress can be thought of as a synchronized and self-controlled cellular state where prooxidants and antioxidants work in harmony to regulate the intensity and duration of redox signaling. The majority of the enzymatic antioxidants are encoded by ARE-driven genes, with these being transcriptionally regulated by Nrf2 [19].

**Figure 1:**
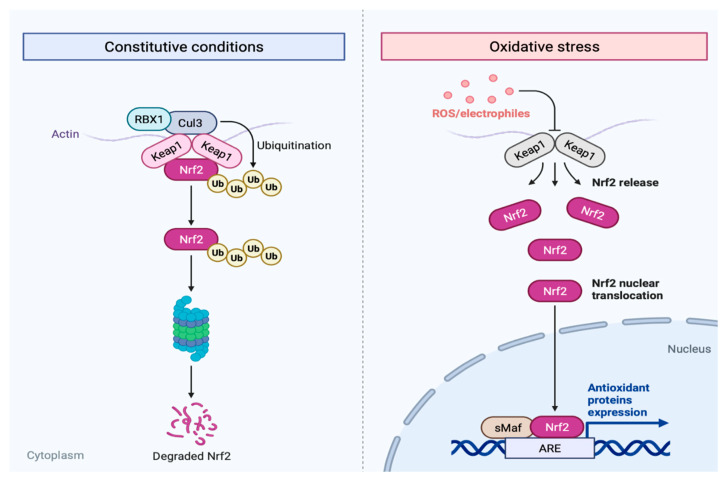
Schematic picture of Nrf2 under constitutive (**left**) and oxidative stress conditions (**right**).

In 1994, Moi et al. successfully isolated and identified a new clone with a high homology to the p45 subunit of the nuclear factor erythroid 2 (NF-E2), which consists of a basic leucine zipper (bZip). The new clone, named NF-E2-related factor 2 (Nrf2), was proven to be a strong activator of RNA polymerase II holding a bZip domain [20]. Based on their binding partners, bZip transcription factors are grouped into different classes; Nrf2 (member of the cap’n’collar (CNC) subfamily) together with NRF1, NRF3, NF-E2, Bach1, and Bach2 [21,22]. Since its discovery, Nrf2 has been a game-changer in cell signaling, where it regulates the expression of more than 250 genes. The majority of its targets are antioxidants and metabolic phase I, II, and III enzymes. Therefore, Nrf2 is mainly known for its cytoprotective role against oxidative and xenobiotic stress (Figure 1). In addition, Nrf2 is also involved in lipid, carbohydrate, and iron metabolism, protein homeostasis via autophagy and proteasomal degradation, DNA repair, and transcriptional and anti-apoptotic regulation [23,24]. Encoded by the *NF-E2-like 2* (*NFE2L2*) gene, Nrf2 is constitutively expressed in all tissues, showing higher expression levels in the brain, kidney, muscle, lung, heart and liver [20,25]. 

As Nrf2 has a prominent role during cellular detoxification, non-stress conditions promote the continuous degradation of this protein. Nrf2 has a half-life of 20 min under normal physiological non-stressed conditions, and its activation level is low [26]. This redox-sensitive negative regulation is primarily secured by Kelch-like ECH-associated protein 1 (Keap1). The main regulation of Nrf2 is via the Nrf2/Keap1-signaling pathway. Upon cytosolic Keap1 saturation, low levels of the newly synthesised Nrf2 maintain the basal expression of its targets [27]. Under these physiological conditions, finer regulation of basal Nrf2 signaling is mainly assured at the nucleus level by β-transducin repeat-containing protein (β-TrCP) in a redox-independent way. Other proteins that can bind to Nrf2 and affect its function and expression of its downstream genes include retinoid X receptor alpha (RXRα), which is involved in differentiation and developmental processes [28], and small musculoaponeurotic fibrosarcoma (sMafs), which is involved in the expression of antioxidant- and phase-II-detoxifying enzymes [29].

There are also other mechanisms involved in Nrf2 regulation. It was shown, for example, that *Nrf2* transcription could be regulated by *Kras*, *Braf,* and *Myc* [30] and, at the translational level, by interaction of the 5′ untranslated region of Nrf2 transcript with the internal ribosomal entry site (IRES) that is involved in the initiation of protein synthesis at ribosomes [31]. The activity of Nrf2 can be modulated by post-translational changes. The main post-translational regulation of Nrf2 is phosphorylation, and several studies have identified GSK-3, MAPKs, PI3K and PERK as the main kinases regulating the phosphorylation status of Nrf2 [32]. There are also number of miRNAs that regulate Nrf2 protein expression levels, and these are the so called redox miRs [33], e.g., miR-144 and miR-28, which are involved in the degradation of *Nrf2* mRNA [34,35]. 

Nevertheless, Nrf2 upregulation is not linear and has not been fully characterised yet. For instance, out of the 605 amino acids that compose human Nrf2, 17% of the residues hold phosphorylation potential (65 serines, 27 threonines, and 10 tyrosines), turning Nrf2 into an intersection point and target of multiple signaling networks [36]. This 67 kDa transcription factor is composed of seven highly conserved domains, which are known as Nrf2-erythroid cell-derived proteins with a CNC homology (Neh). The bZip is located at the Neh1 domain and is vital for Nrf2 to interact with its DNA targets. The remaining domains (Neh2-7) exert protein–protein interactions with activators and inhibitors, influencing Nrf2 activity and assuring its post-transcriptional regulation [37,38]. Nrf2 has several binding partners that can affect its function and expression of downstream genes, including sMafs. There are different sub-types of sMafs, and they are mainly involved in the expression of antioxidant- and phase-II-detoxifying enzymes [39].

As with other CNC transcription factors, Nrf2 forms bZip–bZip heterodimers with the small-Maf bZip subfamily, which comprehends MafF, MafK, and MafG [21,40,41,42]. Specifically, the leucine zipper is responsible for hydrophobic heterodimerization, while the positively charged basic region interacts with the negatively charged phosphate backbone of DNA to determine the binding specificity [38,43,44]. sMaf peptides recognize 13 bp or 14 bp Maf recognition elements (MARE). These palindromic DNA consensus sequences (5′ TGC-core-GCA-3′) are divided into CRE-MARE and TRE-MARE types. The first comprises an 8 bp cAMP-responsive element (CRE) core (TGACGTCA) that is recognised by the CRE binding protein (CREB). The former type carries an activator protein-1 (AP-1) binding site as a core. These AP-1 sites hold a 7 bp consensus sequence (TGA(G/C)TCA) and, being regulated by 12-O-tetradecanoylphorbol-13-acetate (TPA), they are also referred to as TPA-responsive elements (TREs) [22,44,45,46].

Direct Nrf2 targets exhibit a recognition motif in their promoter region known as antioxidant-response elements (AREs), which are also termed electrophile-responsive elements (EpREs). The consensus sequence (5′-TGA(G/C)NNNGC-3′) of these cys-acting elements contains a section with a high similarity to a partial TRE/AP-1 sequence (TGA(G/C). While large Maf peptides form homodimers that bind to both GC dinucleotides flanking MARE sequences, sMaf peptides complexed with Nrf2 bind to the GC-3′ dinucleotide of ARE/EpRE, allowing the transcription factor to recognize the TRE/AP-1 half-site of ARE motifs [44,46]. Neither a single Nrf2 molecule nor an Nrf2 homodimer binds successfully to these DNA motifs without sMaf peptides, making them essential partners of Nrf2 [22,42]. Hence, sMafs mediate the interaction between Nrf2 and DNA, allowing Nrf2 to regulate the expression of ARE-driven genes. Homo- and heterodimers exclusively of sMaf peptides are a possibility, but since sMafs lack transactivation domains, these dimers act as competitors of Nrf2, acting as transcriptional repressors [22,40,44,46].

### Brief Nrf2 Structural Regulation

Located at the N-terminal, Neh2 (amino acids 16-86) is a negative regulatory domain as it holds Keap1-recognition sites [38,47]. Structurally, Keap1 as well as the N-terminal broad complex, Tramtrack, Bric-a-Brac (BTB), and the C-terminal double glycine repeat (DGR) domains are linked by an intervening region (IVR) [48]. The BTB domain is crucial for Keap1 homodimerization [49] and Cullin 3 binding, an element of Cullin-3-based E3 ubiquitin ligase complexes [50]. The DGR domains (also known as Kelch domains) of Keap1 homodimers bind to the DLG and ETGE motifs of Neh2 with a low and high affinity, respectively. When this configuration is achieved, seven lysin residues situated between DLG and ETGE become properly aligned for Nrf2 ubiquitination [50,51,52]. Additionally, the DGR domains are also responsible for Keap1 interaction with actin filaments, retaining Nrf2-Keap1 complexes in the cytosol and blocking Nrf2 transcriptional activity [48].

The regulation exercised by Keap1 is redox-dependent. In humans, Keap1 possesses a total of twenty-seven cysteine residues on its structure. Some function as electrophilic sensors; cysteines surrounded by basic amino acids have a lower pKa, facilitating the deprotonation of the thiol group into thiolate anion, which can be reversibly oxidised into sulfenic acid. Hence, oxidative stress induces cysteine oxidation, forcing Keap1 to undergo conformational changes that prevent Nrf2-Keap1 complex formation [38,53]. One of the most-known hyper-reactive cysteines of Keap1 is Cys151, which is located at the BTB domain and is flanked by the basic residues His129, Lys131, Arg135, Lys150, and His154 [51]. 

Moreover, activated by mTORC1-dependent phosphorylation at Ser351, p62 interacts with the Nrf2-binding site of Keap1 and competitively inhibits Nrf2-Keap1 interaction, inducing, as a result, Nrf2 accumulation and activation [54]. A key role in this cellular response is played by p53. This is a transcription factor activated by DNA damage, which regulates the expression of several target genes, leading to cell cycle arrest aimed at allowing time for the repair of DNA damage. One of the p53 target genes, p21, interacts with Keap1 and inhibits Nrf2 ubiquitylation and degradation. In contrast, activated p53 alone acts as a suppressor of Nrf2, but when the p53 amount increases in the cytoplasm, MDM2 promotes its ubiquitylation and degradation by the proteasome, thus protecting the accumulation and activation of Nrf2. Finally, IKKβ acts as an activator of Nrf2, where it increases the amount of free Nrf2 by competing directly with it for binding sites on Keap1 [55].

The p62-NRF2 relationship is pivotal in cellular responses to oxidative stress. Acting as an adaptor protein, p62 stabilizes Nrf2 by disrupting its interaction with Keap1, resulting in the nuclear translocation of Nrf2 and subsequent activation of genes involved in cytoprotecting and antioxidant defense. Dysregulation of this pathway, such as impaired p62 function or sustained Nrf2 activity, has been implicated in various diseases, including tumorigenesis and neurodegenerative disorders, underscoring the importance of understanding and targeting this intricate interplay [56,57].

The Neh4 (amino acids 112-134) and Neh5 (amino acids 183-201 aa) domains, together with the C-terminal Neh3 domain (amino acids 562-605), are the transactivation domains of Nrf2 [37,38]. Particularly, Neh3 is the binding domain for the chromo-ATPase/helicase DNA binding protein 6 (CHD6), which has been proven to be indispensable for Nrf2 transactional activity [58]. CREB is a typical example of a co-activator that binds separately to both Neh4-5 domains in a synergistic manner [59]. Although they are independent domains, both Neh4 and Neh5 have been reported to interact with other co-activators, such as the mediator of RNA polymerase II transcription subunit 16 (MED16) [60], a catalytic subunit of the chromatin-remodeling complex (BRG1) [61], and the receptor-associated coactivator 3 (RAC3) [62]. Both domains also bind to co-repressors, like the silencing mediator for retinoid- or thyroid-hormone receptors (SMRT) [63] and the glucocorticoid receptor (GR) [64], which promote histone deacetylation. Thus, the Neh4-5 transactivation function varies depending on the target gene. 

**Figure 2:**
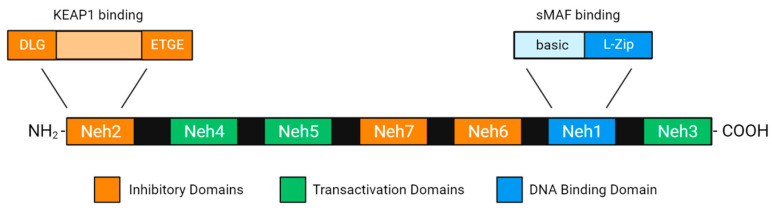
Brief schematic presentation of the Nrf2 structure.

Both the Neh7 (amino acids 209-316) and Neh6 (amino acids 338-388) domains are responsible for Nrf2 suppression in a Keap1-independent manner [37,38]. Being the last domain to be identified, Neh7 interacts with the DNA-binding domain of retinoic X receptor alpha (RXRα), inhibiting the recruitment of co-activators to the promoter region of target genes [65]. Similar to Neh2, the Neh6 domain also contributes to Nrf2 negative regulation via protein ubiquitination. This suppression is mediated by βTrCP, which possesses an F-box domain that interacts with S-phase kinase-associated protein 1 (Skp1), serving as an adaptor of Skp1-Cullin1-Roc1/Rbx1 (regulator of Cullin1/RING-box protein 1) E3 ubiquitin systems. Thus, through the DSGIS and DSAPGS motifs, Neh6 couples to βTrCP, resulting in Nrf2 ubiquitination [66]. Hence, while Neh2 is necessary for primary cytosolic Nrf2 inactivation (Keap1-mediated), Neh6 is imperative for the main nuclear Nrf2 degradation pathway (βTrCP-mediated). A schematic picture of the Nrf2 structure is presented in Figure 2.

The activity of DSGIS is enhanced after phosphorylation by glycogen synthase kinase 3 (GSK3), an important regulator of cell fate and metabolism [66]. GSK3 is activated after the phosphorylation of tyrosine residues (Tyr279 of GSK3α or Tyr216 of GSK3β) and is repressed after the phosphorylation of serine residues (Ser21 of GSK3α or Ser9 of GSK3β) [67]. Different kinases influence GSK activity, both directly and indirectly. For example, by converting phosphatidylinositol biphosphate (PIP2) (4,5) into phosphatidylinositol (3,4,5) triphosphate (PIP3), phosphatidylinositol 3 kinase (PI3K) directly activates 3-phosphoinositide-dependent kinase 1 (PDK1), which in turn activates protein kinase B (PKB or Akt), resulting in the phosphorylation of GSK3, blocking its activity, and indirectly promoting Nrf2 signaling, where Nrf2 is translocated into the nucleus to bind the ARE region and upregulate a series of downstream cryoprotective genes [68]. By reverting PIP3 to PIP2, the phosphatase and tensin homolog (PTEN) interrupts PI3K-PDK1-Akt signaling, promoting GSK-3β activity and consequently promoting Nrf2 suppression. Moreover, evidence shows that cAMP-dependent PKA [69] and certain PKC isoforms [70,71] are also capable of GSK3 inhibitory phosphorylation. Nevertheless, the role of these complex phosphorylation cascades and their multiple effectors and regulators in Nrf2 signaling is not straightforward and cannot be restricted to GSK3 signaling. 

**Figure 3:**
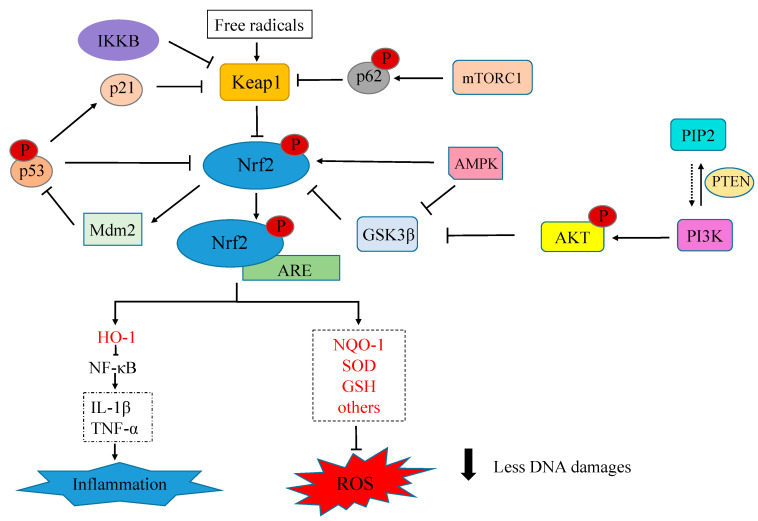
Regulation of Nrf2 by different proteins involved in different signaling pathways.

As mentioned, localized between Neh6 and Neh3, Neh1 (amino acids 434-561) is the DNA-binding domain of Nrf2. Additionally, a nuclear localization signal (NLS) and a leucine-rich nuclear export signal (NES) motif overlap the basic and leucine zipper sites of Neh1. NLS is exposed after Neh2 is released from Keap1, allowing the translocation of Nrf2 from the cytosol to the nucleus [72]. Adenosine-monophosphate (AMP)-activated kinase (AMPK), which is induced under energy-deficient conditions, phosphorylates and inactivates NES, promoting Nrf2 nuclear accumulation and ARE-mediated transcription. Additionally, AMPK phosphorylates and inhibits GSK3β, a promoter of Nrf2 nuclear export [73]. When activated after GSK3β-mediated phosphorylation, Fyn phosphorylates and activates NES, contributing to Nrf2 nuclear escape [74]. Furthermore, the E2 ubiquitin-conjugating enzyme UbcM2 can bind to an intact site of Neh1, stabilising and activating Nrf2 directly in the nucleus. Regulation by UbcM2 involves a non-catalytic cysteine (Cys-136) and an active-site cysteine (Cys-145), with the first one being sensitive to the cellular redox status [75]. As discussed above, the Nrf2-Keap1 pathway can interplay with other pathways such as MAPK, PI3K/Akt, and the Wnt-signaling member (GSK3). A summary of Nrf2 regulation and its interaction with some other pathways is presented in Figure 3 below. 

This review describes the role of Nrf2 in non-pathogenic and pathogenic cells. 

## 2. Role of Nrf2 in a Non-Pathogenic Setting with a Focus on Cellular Responses to High Levels of ROS, e.g., Those Induced by Ionizing Radiation

Ionizing radiation used in radiotherapy is known to kill cancer as well as healthy normal cells partly by the induction of ROS and the production of DNA damage as consequence. A protective role of the Nrf2-ARE pathway against the induction of DNA damage [76,77,78], for example that induced by exposure to ionizing radiation [79], has been reported. Radiation can also accelerate the senescence process of the exposed surviving cells and affect the ability of the exposed healthy stem cells to differentiate. Healthy stem cells with the capacity to differentiate to different linages are needed for replacing radiation-induced damaged tissue with normal healthy tissue. In the non-pathogenic part of this review, we focus on the role of Nrf2 signaling in DNA damage/repair, its role in the differentiation process of stem cells (adipogenesis, neurogenesis, and osteogenesis as examples), and its role in senescence.

### 2.1. The Role of Nrf2 in DNA Repair and the Promotion of Survival after Exposure to Ionizing Radiation

Intracellular ROS can be formed endogenously as natural by-products, for example from the mitochondrial electron transport chain, or exogenously, for example through exposure to ionizing radiation. In oxidative stress conditions, when the levels of ROS exceed the cellular antioxidant capacity, ROS can react with DNA molecules and induce single-strand breaks (SSBs) and oxidative base damage as well as double-strand breaks (DSBs) [80]. SSBs and base damage are mainly repaired by the base excision repair system (BER), in which DNA glycosylases recognize and remove damaged DNA bases, resulting in a gap. The gap is then filled with new a DNA base by DNA polymerase and sealed by DNA ligase. Several glycosylases with the ability to remove oxidative base damage have been identified in both bacteria (the proteins MutY and MutM) and higher organisms, e.g., the proteins OGG1 and OGG2 [81]. DSBs can be repaired mainly by non-homologous end joining (NHEJ) and homologous recombination (HR) [82]. NHEJ is active throughout the cell cycle and allows rapid joining, and homologous recombination (HR) is active in the G2-S phase when a sister chromatid is present and allows reliable and error-free repair. HR repair is possible if DNA resection is induced after DSB. The generated single-strand regions of DNA during the resection result in the binding of RAD51 protein filaments to DNA, mediating homology-directed strand invasion by BRCA2 [83]. When the resection exposes complementary sequences, repair can be achieved through RAD52-mediated single-strand annealing. Recognition of DSBs by either KU70/KU80 or poly ADP-ribose polymerase (PARP) leads to canonical NHEJ and alternative NHEJ, respectively. Prior to repair, DNA damage is detected by sensing proteins such as ATM (ataxia telangiectasia mutated), ATR, DNA-PK (DNA-dependent protein kinase), and PARP [84]. They are constitutively transcribed proteins that quickly undergo post-translational activation as a consequence of DNA damage and replication errors [82,85]. ATM binding and activation lead to the phosphorylation of many other target proteins (53BP1, H2AX, CHK2, SMC1, TP53, BRCA1, etc.), which are implicated in DNA damage response, i.e., DDR. DNA-PK is a trimeric nuclear serine/threonine kinase made up of a catalytic subunit and two DNA-targeting proteins, KU70 and KU80 [86,87]. In contrast to ATM, ATR is thought to primarily process single-strand DNA (ssDNA) breaks and tends to be enrolled on replication protein A (RPA)-coated ssDNA [88]. However, several reports have indicated that ATR can also respond to IR-induced DNA breaks [89,90]. The assembly of the ATR complex at DNA breaks activates signaling that regulates the cell cycle, DNA repair, and DNA replication. CHK1-CDC2, which manages cell cycle transitions, is primarily dependent on ATR activation [91,92]. DSBs are considered to be the most critical and deleterious lesions for cells. DSBs, if not repaired, can cause chromosomal aberrations or mutations that can lead to a loss of genetic material, resulting in cell death. Cells use different mechanisms to resist the deleterious effects of, for example, DNA-damaging agents such as radiotherapy and chemotherapy. The mechanisms include the elevated expression of antioxidants that can reduce intracellular ROS before they react with DNA and an effective DNA damage signaling and repair that can repair the damage so that the cell may survive.

There are few studies indicating the role of Nrf2 in DNA damage signaling and repair. As an example, it was shown that the transcriptional regulation of 53BP1 can be regulated by Nrf2 [77]. Some studies have also reported the involvement of Nrf2 in the repair of oxidative DNA base damage and its contribution to the pro-survival response after exposure to ionizing radiation [93]. In the work carried out by Singh et al., the antioxidants vitamin C and butylated hydroxyanisole (BHA) were used to suppress estrogen-metabolism-mediated oxidative DNA damage, which is known to decrease levels of Nrf2 [94]. Their results showed that the antioxidant treatment prevented the E2-mediated damage and significantly increased the levels of Nrf2 expression. Furthermore, they demonstrated that the oxidative damage was reduced through the Nrf2-dependent regulation of OGG1 which is the main DNA glycosylase enzyme involved in the removal of 8-oxo-dG, a marker of oxidative stress [76,95,96]. Also, the protective effect of oyster (Ostrea plicatula Gmelin) polysaccharides (OPS) was assessed because of their antioxidant activity [97]. It was shown that OPS decreased erythrocyte micronuclei (MN) formation as well as bone marrow toxicity induced by mutagens by increasing the Nrf2 nuclear level [97]. An accumulating number of recent studies have indicated that quercetin has powerful antioxidant and free-radical-scavenging properties. A report illustrated that quercetin can be used to reduce the effect of 1,2-dimethylhydrazine (DMH), a toxic environmental pollutant and a colon-specific carcinogen [84]. Quercetin supplementation potently attenuated 8-oxo-dG production and decreased the levels of AP sites. Concomitantly, quercetin reversed the DMH-induced expression pattern of the Nrf2 pathway by overexpressing Nrf2 and decreasing Keap1 levels. Thus, quercetin successfully suppressed DMH-induced DNA damage through modulating the Nrf2/Keap1-signaling pathway [98].

In another study, mangiferin, a natural antioxidant, was demonstrated to play a protective role against DNA damage induced by etoposide, a chemotherapy drug. The antioxidant mangiferin effectively inhibited etoposide-induced DNA damage in terms of MN formation and DNA strand breaks [99]. This could be due to an increased nuclear accumulation of Nrf2 after mangiferin treatment. Moreover, NQO1, an Nrf2-signaling target involved in PARP activity [99], was significantly upregulated by mangiferin treatment [100]. Furthermore, a protective effect of 18α-GA, a bioactive triterpenoid, has been shown to activate Nrf2 against mitomycin C (MMC), a chemotherapeutic agent inducing DNA damage. The results showed a decreased level of DNA damage with 18α-GA pre-treatment and suggested that Nrf2 activation by 18α-GA is regulated through the MAPK (ERK1/2) pathway [101].

Srivastava et al. showed that buthionine sulfoximine (BSO) treatment depleted glutathione (GSH) in mice and induced a high expression of Nrf2 [102]. When this treatment was combined with arsenic, it resulted in in vivo genotoxicity due to the generation of ROS in GSH-depleted mice and thus caused abnormal metaphases and different chromosomal aberrations. To limit this genotoxicity, they demonstrated that a pre-treatment with N-acetyl-L-cysteine (NAC) promoted a significant intracellular induction of GSH, which, in turn, could cope with increased ROS levels [102].

In another study, Jayakumar et al. showed that when Nrf2 was inhibited or silenced in cancer cells, a significant slowdown in DNA repair occurred [78]. They found that Nrf2′s influence on DNA repair was independent of ROS levels. Furthermore, Nrf2 could regulate HR by controlling the mRNA level and foci formation of RAD51 in an ROS-independent manner [78,93]. This suggests that Nrf2 may promote HR during DSB repair. Sun et al. also showed that the Nrf2 protein level was markedly increased in cells with DSBs. In addition, their study showed that the depletion of Nrf2 decreased the percentage of BRCA1 and RAD51 foci-positive cells, which are indicators of HR efficiency [103]. Another report also demonstrated that Nrf2 promoted G2 cell cycle arrest by directly influencing ATR phosphorylation. This subsequently activated the ATR–CHK1–CDC2 signaling cascade, which is ROS-independent [104]. They showed that the downregulation or silencing of Nrf2 caused the transcriptional repression of both ATM and ATR expression and led to aberrant or insufficient DDR signaling and a higher cytotoxicity to cisplatin. They indicated a crosstalk between the antioxidant-response (AR) and DDR pathways that extended the scope of action of Nrf2 in promoting therapeutic cancer resistance [104]. This could be directly achieved by Nrf2 binding to the promoter regions of ATM and ATR that, in this case, repressed their expression. On the other hand, Nrf2 could act indirectly by its downstream proteins, which could in turn regulate ATM and ATR transcription. Furthermore, upon Nrf2 activation by bardoxolone methyl, a semi-synthetic triterpenoid, cells showed less radiation-induced DNA damage in the S and G2 phases of the cell cycle due to the promoting role of Nrf2 in the HR pathway [77]. The Nrf2 transcription factor mainly regulates the expression of a wide range of genes that code for antioxidants and other proteins responsible for the detoxification of xenobiotics and ROS. The gain of functions of Nrf2 may protect cells from toxicity induced by ionizing radiation, but additional mechanisms may also be involved, linking Nrf2 to radioresistance [77,93]. 

**Figure 4:**
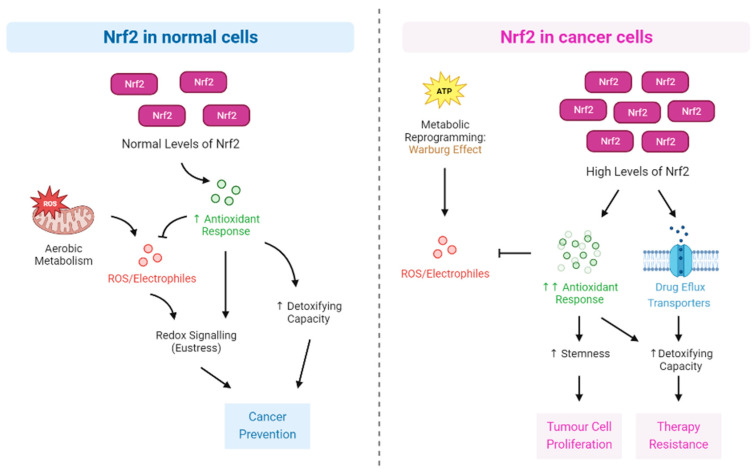
Nrf2 in normal (**left**) and cancer (**right**) cells. In normal cells, Nrf2 signaling is highly upregulated, with Nrf2 being mainly activated for cell protection. Under this regulated environment, Nrf2 fights the intracellular ROS inadvertently produced during aerobic metabolism by promoting the gene expressions of different antioxidants. The antioxidant response is vital for detoxifying cells with toxic ROS levels, avoiding distress conditions where ROS-induced mutagenic events can occur. Apart from that, the fine balance of ROS by antioxidants allows for a synchronized and self-controlled eustress state, where prooxidants and antioxidants work in harmony to regulate the intensity and duration of redox signaling. Thus, under normal conditions, the antioxidant response controlled by Nrf2 is key in cancer prevention. However, many cancer cells have constitutive and dysregulated activation of Nrf2. In this scenario, higher Nrf2 levels combat intracellular ROS of anaerobic metabolism due to the Warburg effect. In addition, in many cancer cells with Nrf2 upregulation, Nrf2 has been implicated in the activation of drug efflux transporters, having, in general, a major role in cell cancer cell detoxification. Additionally, cancer cells take advantage of the antioxidant role of Nrf2 to keep ROS at minimum levels by transiting to a stem state. Stemness, antioxidants, and efflux transporters confer great resistance to the main therapies currently practiced in the fight against cancer, namely chemo- and radiotherapy. Hence, in cancer cells, Nrf2 signaling is vital for tumor survival after treatment and its subsequent repopulation due to Nrf2-induced stemness properties.

Notably, cancer cells possess heightened levels of ROS compared to normal cells, which is attributed to factors such as oncogene mutations and mitochondrial damage [105,106] and their metabolism. In the context of cancer cells, Nrf2 assumes an antioxidant role that can exhibit either protective or detrimental effects based on ROS levels. For instance, Nrf2 activation in cancer cells can promote cell survival and redox homeostasis, whereas excessive Nrf2 activity may contribute to tumor progression [30,107]. Figure 4 illustrates the role of Nrf2 in normal and cancer cell survival and therapy resistance.

### 2.2. Role of Nrf2 in Stem Cell Differentiation

In recent years, scientists have shown that Nrf2 is involved in stem cell differentiation processes. Nrf2 is highly expressed in undifferentiated embryonic stem cells compared to differentiated cells. Notably, Nrf2 is also highly expressed in cancer stem cells as compared with differentiated cancer cells [108]. In addition to regulating the cellular redox balance, Nrf2 has been considered to control several cellular processes such as stem cell proliferation and lineage-specific differentiation. Osteoblasts and osteoclasts are two indispensable elements involved in bone homeostasis, where ROS mediate physiological signaling. In this context, Nrf2 plays a major role. It is widely known that osteoclast differentiation is highly regulated by the intracellular level of ROS. When Nrf2 activity is reduced, the ROS level increases and thus osteoclast differentiation takes place [109]. 

**Figure 5:**
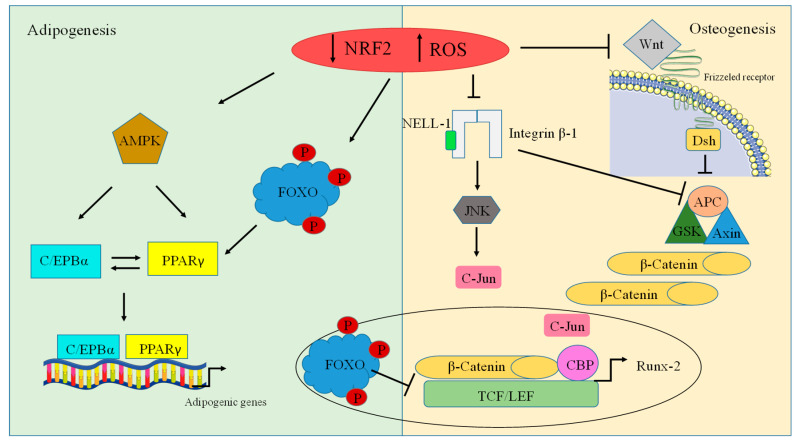
The role of Nrf2 and ROS in adipogenesis and osteogenesis. ROS suppresses important signaling pathways necessary for bone formation while simultaneously stimulating pathways that promote the formation adipocytes. The Wnt/βeta catenin and NELL-1 pathways play a key role in promoting osteogenesis, but their activity is hindered when Nrf2 levels are low and ROS levels are high. Consequently, ROS can trigger the phosphorylation of FOXO proteins, causing them to translocate into the nucleus and hinder the signals that promote bone formation, leading to a shift towards adipogenesis.

The activity of osteoblasts and osteoclasts is positively regulated by the Wnt/β-catenin-signaling pathway. Wnt/β-catenin signaling plays a crucial role in inducing osteogenesis and is initiated when extracellular Wnt ligands bind to frizzled receptors that have seven transmembrane domains (Frz). This leads to the stimulation of a cytoplasmic phosphoprotein, disheveled (Dsh), which acts by inhibiting the axin, glycogen synthase kinase 3 (GSK3), and adenomatosis polyposis coli (APC) protein complex [110]. When Wnt signaling occurs, the Axin/APC/GSK3 complex stops β-catenin degradation, resulting in β-catenin being moved from the cytoplasm to the nucleus [111]. In the nucleus, β-catenin binds to T-cell factor/lymphoid-enhancing factor (Tcf/Lef), which acts as a transcriptional effector that stimulates the activation of Wnt target genes such as Runx2, thus stimulating osteogenesis. Moreover, on the one hand, neural-epidermal-growth-factor-like 1 protein (NELL-1) binds to integrin β1, whereas on the other hand, it increases β-catenin nuclear localization, which increases the transcription of Runx2 and Osterix. NELL-1 activates ERK1/2 and JNK, which phosphorylate and activate Runx2 [112]. High levels of ROS can inhibit the signaling pathways of Wnt/catenin and NELL-1, thus inhibiting osteogenesis by stimulating a group of the Forkhead family of the transcription factor (FOXO) protein to undergo phosphorylation and migrate to the nucleus [112], where they decrease the expression of genes that induce osteogenesis. On the other hand, this stimulates adipogenic differentiation by activating peroxisome-proliferator-activated receptor gamma (PPARδ), which is a nuclear receptor that plays a major role in lipid metabolism and adipogenic differentiation [113]. Another way of activating adipogenesis is through the direct activation of CCAAT-enhancer-binding protein beta and alpha, C/EBPβ and C/EBPα, which are major adipogenesis regulators during the early and terminal phases of differentiation. Upon low levels of Nrf2, AKT is stimulated and activates C/EBPα and PPARγ. The coordinated activity between these two transcription factors acts as a positive feedback loop, in which PPARγ can activate the promoter of the gene encoding C/EBPα and vice versa [114] in order to regulate the transcription and expression of other adipogenic-specific genes (Figure 5). 

On the other hand, it has been reported that the activation of AKT stimulates the activation of Nrf2 downstream, where it promotes osteogenic differentiation. Xiong et al. showed that curcumin enhances the protein level of Nrf2 and plays a crucial role in the curcumin-induced osteogenic differentiation of human periodontal ligament stem cells [115]. In another study by Xue et al., a new binding partner of Nrf2, optineurin, was identified. This molecule interacts with Nrf2 and was shown to be a negative modulator of osteogenesis. In vivo mouse studies showed that a deficiency in Nrf2 significantly impairs bone formation and reduces bone volume [116]. This shows the important role of Nrf2 in postnatal bone development. A recent study showed that knocking down Nrf2 resulted in an increase in ROS formation and repressed the tendency of osteogenic differentiation in periodontal ligament stem cells. This was mediated by the application of ochratoxin A, an Nrf2 inhibitor that blocks osteogenic differentiation ability in the early as well as in the late passage of MSC, suggesting the vital role of Nrf2 in cyclic-mechanical-stretch-induced osteogenic differentiation in periodontal ligament stem cells (PDLSCs), which might be associated with the expression of antioxidants and controlling the production of ROS [117].

It is important to highlight the crucial role of Nrf2 in maintaining mesenchymal stem cell (MSC) stemness. Yoon et al. pointed out a link between Nrf2 and Sirtuin (SIRT) expression in stimulating the self-renewal and differentiation of MSCs. They compared Nrf2 activity in the early and late passage of cells, demonstrating that the inhibition or induction of Nrf2 nuclear localization has a stimulated colony-forming ability as well as an ability to generate the proliferation of MSCs. Upon treating these cells with both an Nrf2 activator (T-BHQ) and an inhibitor (OTA), the in vitro osteogenic differentiation potential was decreased [118]. There are conflicting results regarding the role of Nrf2 in osteogenic differentiation. Human periodontal ligament cells can be efficiently differentiated to the osteogenic lineage by increasing Nrf2 levels in nuclear extracts. Nrf2-knockout mice showed a significant deficit in postnatal bone acquisition [119]. Taken together, Nrf2 is a key factor in MSC maintenance and osteogenesis differentiation. When Nrf2 is lacking, MSCs cannot self-renew and differentiate into the osteogenic lineage [118].

Many researchers have focused on the implication of Nrf2 in adipocytes. Various studies have demonstrated that Nrf2 could inhibit adipogenic differentiation in preadipocytes. This is due to the fact that specific transcription factors drive MSCs into adipocytes. Recent data have shown that, both in the bone marrow and in C3H10T1/2 mesenchymal stem cells, an Nck1 deficiency activates the PDGFRα-Nrf2 anti-adipogenic signaling pathway by interacting with tyrosine-phosphorylated PDGFα through its SH2 domain of Nrf2, leading to the activation of Nrf2, which, upon nuclear translocation, induces the expression of the antioxidant genes *Nqo1* and *Hmxo1* as well as *Pdgfa*, which encodes PDGF-A, a specific ligand for PDGFRα. This signaling pathway impairs the differentiation ability of adipocytes by limiting body fat accumulation [120]. Another report demonstrated that different chemical activators of Nrf2 such as SFN or butein inhibit lipid accumulation by decreasing the expression of adipogenic differentiation genes such as PPARγ and EBPα and fatty-acid-binding protein 4 [121]. In addition, adipogenesis is inhibited by Nrf2, which activates the aromatic receptor pathway that is responsible for inducing differentiation from 3T3-L1 preadipocytes and MEF to mature adipocytes. These inhibitory effects of Nrf2 are linked to early adipogenesis. On the other hand, Nrf2 knockdown alleviates oxidative-stress-induced lipid accumulation. Systematically, oxidative stress promotes Nrf2 recruitment to the sterol-regulatory-element-binding protein 1 promoter (SREBP1), thus stimulating target gene transcription and consequent lipogenesis [122]. While several studies have demonstrated that Nrf2 may function as a negative regulator in adipogenesis, contradictory results have also shown that Nrf2 plays a crucial role in increasing the differentiation capacity of preadipocytes [123]. 

Notably, it was shown that an excessive metabolism of palmitic acid (PA) was associated with ER stress in cells [124]. It was also shown that the treatment of HK-2 cells with PA induces the accumulation of lipid in the cells, resulting in a lower cell viability and lipotoxicity. Higher levels of mitochondrial ROS production and mitochondrial dysfunction were also observed in PA-treated HK-2 cells [125]. In parallel, activation of the Nrf2/ARE pathway and the upregulation of its important target enzymes HO-1 and NQQ-1 was also reported, indicating that hyperlipidemia by PA in non-adipose cells/tissues results in Nrf2/ARE activation [125]. 

Recent reports have stated that Nrf2 not only maintains the shape of lipid droplets in adipocytes but also increases the storage of triglycerides as well as stimulating the *PPARγ* gene that plays a key role in adipogenesis, where it regulates the development of preadipocytes as well as mature adipocytes. Pi et al., demonstrated that wild-type mice showed an expansion of fat pads and an increase in white adipose tissue weight compared to *Nrf2 KO* mice. Upon the absence of Nrf2, different genes that are responsible for adipogenic differentiation such as PPARγ and CEBPα are downregulated [126]. Finally, Nrf2 plays a critical role in adipose tissue, and the effect of Nrf2 on adipocyte differentiation needs to be interpreted in accordance with systemic metabolic changes. Further studies to elucidate and explain the underlying mechanisms of adipocyte differentiation are required. A schematic picture of Nrf2’s roles in osteogenesis and adipogenesis is presented in Figure 5. 

There is also limited information available showing that the overexpression of Nrf2 in bone marrow stem cells (BMSC) promotes neural differentiation, while the inhibition of endogenous Nrf2 expression by Nrf2 inhibitors hinders this process [127]. Similarly, Nrf2, when combined with a neuropoietic cytokine, CNTF, induces neural stem cell self-renewal and neural development through a signaling molecule, STAT3 [128]. Furthermore, Kärkkäinen et al. reported that the neural differentiation potential of neuronal stem cells (NSC) in embryonic cells was highly activated when the Nrf2 gene or an Nrf2-activating molecule was overexpressed in mice [129]. In addition, Erk1/2 activation and Akt phosphorylation triggers Nrf2, which promotes axonal and nerve growth factors in PC12 cells [130] and neuronal progenitors in the presence of Petrosiol E treatment [131]. Another study described the role of rotenone in the Nrf2 pathway. They suggested that non-/low-cytotoxic or moderate concentrations of rotenone can induce Nrf2 pathway activation by upregulating NQO1, SRXN1, and HO1, thus increasing neural stem cell and astrocyte differentiation [132]. Altogether, these studies indicate a relationship between Nrf2 signaling and the induction of neural differentiation.

Astrocytes, for instance, seem to be the main brain cell type involved in Nrf2-ARE-cascade-mediated neuroprotection [133]. A study revealed that a primary cortical culture derived from ARE-reporter mice displayed selective ARE-promoter activity in astrocytes when treated with sulforaphane and tert-butylhroquinone (Nrf2 activators) for 48 h. This gave rise to neuroprotection against hydrogen peroxide and glutamate toxicity. Also, human U373 astroglial cells secreted higher levels of glutathione and cysteinglycine when treated with these Nrf2 activators, corroborating the neuroprotective role of the Nrf2-signaling pathway in astroglial cells [134].

### 2.3. Nrf2 and Cancer Stem Cell Differentiation

Nrf2 plays a crucial role in the treatment response of several tumour types such as non-small-cell lung cancer, breast cancer, glioma, bladder, hepatocarcinoma, and prostate cancer. It induces a pro-survival tumour environment that boosts tumour growth and upholds cancer cells for chemoresistance and radiotherapy [135]. 

Recently, a significant role was demonstrated for Nrf2 in cancer stem cells by several reports. It was shown that Nrf2 signaling plays a role in maintaining cancer stem cell stemness and increasing self-renewal due to different intracellular and extracellular stress conditions. It was shown that Nrf2 knockdown promotes a great decrease in the antioxidant properties of glioblastoma cancer stem cells, thus decreasing their self-renewal capacity, lowering the number of spheres, stimulating cell differentiation, reducing their proliferative capacity, and increasing their sensitivity to ionizing radiation [108]. Another report pointed out that Nrf2 overexpression in breast cancer stem-like cells is associated with GSH biosynthesis that stimulates the signaling pathway of the glutamate cysteine ligase catalytic subunit and, subsequently, a reduction in intracellular ROS accumulation in these cells, thus keeping their stemness properties [136]. Additionally, CD133, a cancer stem cell marker of colon cancer, mediates a signaling pathway through the activation of 3-kinease/serine-threonine kinase (PI3K/AKT), resulting in an increase in Nrf2 protein levels and increasing sphere-formation capacity and proliferation, thus highlighting the significant role of the Nrf2 pathway in sustaining CSC-like properties [137]. Interestingly, a higher proportion of cancer stem cells in tumour masses has been associated with therapy resistance and relapse [138,139,140]. The role of Nrf2 in a non-pathogenic setting is summarized in Table 1.

### 2.4. Nrf2 and Cell Senescence

The modulation of the cellular localization of Nrf2 is very important to prevent MSC aging during prolonged cell passages in vitro, since oxidative stress prompts the premature senescence of MSCs. Nrf2 expression protects MSCs from cell death and apoptosis caused by oxidative stress and retains their proliferation ability [141]. ROS can cause severe damage to the genome and speed up telomere shortening, which is a significant signaling pathway involved in maintaining the senescence phenotype [142]. The mechanism of action of Nrf2 consists of a network of multiple genes at the protein level that modulate and regulate senescence.

For instance, protein kinase has an essential role in maintaining homeostasis in an intracellular environment. Not only does it play a role in energy metabolism, but it also induces cell cycle arrest by subsequently phosphorylating P53 and P21. AMPK has also been shown to reduce oxidative stress, which can extend the healthy life span of a cell and prevent cell senescence [143]. Several natural compounds have been widely described to prevent cellular senescence and aging-related diseases through AMPK/Nrf2 signaling.

Phlorentin, which belongs to the flavonoid family, promotes the phosphorylation of AMPK at Thr172, which upregulates the expression of both Nrf2 and HO-1, thus preventing oxidative-stress-induced endothelial cellular senescence [144]. Another example is cordycepin, which is a natural derivative of adenosine with multiple pharmacological activities such as anti-oxidation, antitumor, and anti-inflammation, has lately been reported to prevent radiation ulcers by inhibiting cell senescence via the AMPK/Nrf2-signaling pathway in rodents [145]. Zhao et al. showed that Nrf2 exerts an anti-aging function by compensating a-Klotho deficiency. They demonstrated that upon crossing α-Klotho-deficient mice with Keap-knockdown ones, the Nrf2 pathway was highly activated and enhanced the lifespan of the mice, and it induced the expression of antioxidant genes to cope with the high oxidative stress, thus attenuating the aging process [146]. Another study demonstrated that the addition of 2′-fucosyllactose constructively activates the Nrf2-signaling pathway that, in turn, promotes the expression of HO-1 and NQO1 in aging model mice [147].

On the other hand, the PERK/Nrf2 pathway plays a dominant role in oxidative and endoplasmic reticulum stress, which regulates mitochondrial ROS production [148]. PERK activation rapidly reduces protein biosynthesis and promotes the clearance of misfolded proteins and activates Nrf2. When PERK is phosphorylated, Nrf2 dissociates from Keap1, is translocated into the nucleus, and regulates the expression of multiple antioxidant genes that maintain the redox homeostasis balance and restore the stability of endoplasmic reticulum stress proteins, thus promoting cell survival [148,149]. Indeed, a crosstalk between Nrf2 and p21 mediates cell protection, the regulation of oxidative stress, and cell senescence. This is due to the 154KRR motif in p21 that directly interacts with the 29DLG and 79ETGE motifs in Nrf2 to compete with Keap1 for Nrf2 binding, thus activating Nrf2 and preventing its degradation [150]. It has also been reported that p53 inhibits Nrf2 expression and function, thus increasing intracellular ROS levels and inhibiting the proteasomal degradation of p53. Luteolin, for instance, has been demonstrated to upregulate the expression of the p53/p21 pathway, thus inhibiting Nrf2 and stimulating apoptosis in colon cancer [151]. This shows the important interplay between the p53/p21 pathway and Nrf2 in delaying cell senescence and regulating cell survival. Additionally, Nrf2 has been shown to be involved in crosstalk with many cytosolic transcription factors such as the aryl hydrocarbon receptor (AHR) and NF-κB. Upon stimulation of the cell by exogenous and endogenous free radicals, AHR with the cytoplasmic enzyme ARNT translocate into the nucleus, thereby inducing the transcription of CYPs and production of ROS by binding to the xenobiotic response elements (XREs) in the promotors of the target genes [152]. Oxidative stress then stimulates IKK, which activates NF-κB and inhibits the Nrf2 activity by enrolling histone acetylase 3 (HDAC3) to the ARE region to stop ARE gene expression [153]. To restore Nrf2 activity, curcumin [154] and cyanoside-3-o-glucoside could be added to activate the Nrf2/HO-1 pathway and thus increase the expression of detoxification genes to protect cells from inflammation and senescence [155]. This section has been summarised as Table 1.

**Table 1:** Nrf2 in non-pathogenic setting.

Mediator	Role	Impact	Reference
All-trans retinoic acid (ATRA)	Nrf2 inactivation	Slows down DNA repair in cancer cells	[78,93]
Vitamin C and butylated hydroxyanisole	Increase Nrf2 expression	Reduces oxidative DNA damage	[94]
Oyster polysaccharides	Increase Nrf2 nuclear level	Decreases in erythrocyte micronuclei formation and bone marrow toxicity	[97]
Quercetin	Nrf2/Keap1 signaling	Suppresses 1,2-dimethylhydrazine (DMH)-induced DNA damage	[99]
Mangiferin	Increase the nuclear accumulation of Nrf2	Inhibits etoposide-induced DNA damage	[100]
18α-GA	Nrf2 activation through the MAPK (ERK1/2) pathway	Prevents DNA damage by mitomycin C	[101]
Buthionine sulfoximine	High expression of Nrf2 through glutathione depletion	Reduces chromosome aberrations	[102]
Knockout	Nrf2 depletion	Hypersensitivity of cells to ionizing radiation in the presence or absence of reactive oxygen species (ROS)	[103]
Knockdown	Nrf2 inactivation	DNA damage response suppression by down-regulating ATM and ATR, leading to enhanced cytotoxicity	[104]
Knockdown	Nrf2 inactivation	Decreases the antioxidant properties of glioblastoma cancer stem cells	[108]
Curcumin	Increase the level of Nrf2 protein	Induces osteogenic differentiation of human periodontal ligament stem cells	[115]
	Deficiency in Nrf2	Impairs bone formation and reduces bone volume	[116]
Ochratoxin A	Knocking down Nrf2	Increases ROS formation and represses the tendency of osteogenic differentiation in periodontal ligament stem cells	[117]
T-BHQ/OTA	Nrf2 activator/inhibitor	Decreases osteogenic differentiation potential	[118]
Knockout	Nrf2 inactivation	Causes a deficit in postnatal bone acquisition	[119]
Nck1 deficiency	Nrf2 activation	Impairs the differentiation ability of adipocytes by limiting body fat accumulation	[120]
SFN/butein	Nrf2 activation	Inhibits lipid accumulation by decreasing the expression of adipogenic differentiation genes	[121]
Nrf2	Activate aromatic receptor pathway	Inhibits adipogenesis	[122]
Knockdown of Keap1	Nrf2 activation	Enhances and accelerates hormone-induced adipocyte differentiation in mouse embryonic fibroblasts	[126]
	Combination of Nrf2 and neuropoietic cytokine	Induces neural stem cell self-renewal and neural stem cell differentiation during embryonic development	[128]
Pyrrolidine dithiocarbamate/Amyloid beta (Aβ)	Nrf2 overexpression	Prevents a reduction in the neurosphere proliferation of neural stem/progenitor cells in Alzheimer’s disease	[129]
Rotenone	Induce Nrf2 pathway activation	Increases neural stem cell and astrocyte differentiation	[132]
Sulforaphane and tert-butylhroquinone	Nrf2 activation	Increases neuroprotection against hydrogen peroxide and glutamate toxicity	[133]
Sulforaphane and tert-butylhroquinone	Nrf2 activation	Secretion of higher levels of glutathione and cysteinglycine in astroglial cells	[134]
	Nrf2 overactivation	Reduces intracellular ROS accumulation in breast cancer stem-like cells, thereby provoking reductive stress	[136]
CD133	Increase Nrf2 protein level	Increases sphere formation capacity and proliferation in stem cells of colon cancer	[137]
Phlorentin	Upregulate the expression of Nrf2 and HO-1	Prevents oxidative-stress-induced endothelial cellular senescence	[144]
Cordycepin	Signal AMPK/Nrf2	Prevents radiation ulcers by inhibiting cell senescence	[145]
Keap1-knockdown	Nrf2 activation	Extends the lifespan of and dramatically improves ageing-related renal phenotypes in mice	[146]
2’-Fucosyllactose	Nrf2 activation	Promotes the expression of HO-1 and NQO1 in aging model mice	[147]
Luteolin	Nrf2 inhibition	Stimulates apoptosis in colon cancer	[151]
Knockdown	Nrf2 inactivation	Stimulates cardiotoxin-induced oxidative stress and impairs proliferation, thus retarding the regeneration of muscles	[156]

## 3. Role of Nrf2 in a Pathogenic Setting

Intracellular pathogens such as protozoan parasites, viruses, and bacteria may modify the activation of Nrf2 by affecting its post-translational modifications and interfering with different protein complexes and the immune response. Pathogens can induce Nrf2 activation via the involvement of ER stress, toll-like receptors, and PI3K/Akt pathways. In this section, we describe the role of Nrf2 in viral, bacterial, and parasite (malaria and Leishmania) infections as examples.

### 3.1. Nrf2 and Infection

Nrf2 regulates gene expression in response to the oxidative stress imposed by pathogens including aging, inflammation, and tissue damage. One of the primary microbicidal processes, oxidative burst, might be compromised during the early phagocytosis of pathogens, which could successfully establish an infection. Even though infections can lead to oxidative bursts, a number of ROS-derived products act as signaling molecules that eventually result in cellular homeostasis [157,158,159,160]. It is interesting to note that the generation of these radicals has evolved in a coevolutionary pattern between pathogens and host cells [161]. Numerous infections affect post-translational modifications, the interplay between protein complexes, and the immune response, which result in Nrf2 activation, particularly those that arise from intracellular pathogens like parasites, bacteria, and viruses. Different mechanisms, like those involved in the activation of PI3K/Akt, the engagement of toll-like receptors (TLRs), and endoplasmic reticulum stress, may be used by pathogens to stimulate Nrf2 [162].

### 3.2. Mechanisms of Nrf2 Activation in Infection

Nrf2 is activated in innate immune cells like macrophages and monocytes when pathogen-associated molecular patterns (PAMP) interact with pattern-recognition receptors (PRRs). For example, nuclear factor kappa B (NF-B) and the adaptor molecule Myd88 (myeloid differentiation primary response gene 88) activate the transcription expression (TE) of the inducible form of nitric oxide synthase (iNOS/NOS2) in response to the recognition of lipopolysaccharides (LPSs) by TLR4 [148]. The intracellular accumulation of superoxide (O_2_^−^) also triggers via the TLR4–MyD88–NF-κB signal transduction pathway, which activates via the TE of phagocytic NADPH oxidase (NOX2/gp91^phox^) [163]. The NO produced by iNOS reacts with O2^•−^ and generates peroxynitrite anions (ONOO^−^), which ultimately target several thiol-based (S-H) redox systems, particularly the reactive cysteines in Keap1 [24,47,164]. The Nrf2 proteolytic degradation of the 26s proteasome is targeted by Keap1, which is an adaptor for the cullin (Cul)3–RING (really interesting new gene)-box protein (Rbx)1 ubiquitin ligase complex [47]. Additionally, in conditions of oxidative stress, ONNO targets some of Keap1’s reactive cysteines, such as (Cys151) [165], which change Keap1’s tertiary structure and prevent its ability to operate as a ubiquitin ligase and degrade Nrf2 [24,47,164].

To activate the transcription of Nrf2-responsive genes possessing DNA antioxidant-response elements (AREs) in their promoters [24], transcribed Nrf2 travels to the nucleus and binds to sMaf transcription factors, like MafF, MafG, and MafK [166]. It is likely that NF-B directly induces the Nrf2 promoter to activate Nrf2 transcription, which is necessary to maintain Nrf2-dependent gene expression. The integration of Nrf2 activation within various types of cellular stresses is facilitated by other E3 ubiquitin ligase complexes, such as Skp1 (S-phase kinase-associated protein 1), Cul1-F-box (SCF), and β-TrCP complex (SCF^β-TrCP^) [167]. Thus, GSK3 recognises and phosphorylates Nrf2’s Neh6 (Nrf2-ECH homology 6) domain [167]. The phosphorylation of Nrf2 at the Neh6 domain can cause different stress forms via the ubiquitination of Nrf2 via the SCF^β-TrCP^ complex with GSK3 and being degraded through the 26s proteasome [24,167]. Nrf2 could also be affected by Hrd1 as a novel E3 ubiquitin ligase [168]. The 26s proteasome enables the Hrd1-targeted Nhe4-5 domain of Nrf2 to be ubiquitinated and degraded [169]. It is significant to remember that Nrf2 activity is greatly influenced by its transcription and expression rate, which is controlled by Nrf2 itself, NF-B, and clock elements that exert a circadian regulation on Nrf2 activity [170].

### 3.3. Nrf2 and Parasitic Infection

Along with infections caused by *Leishmania* spp., Nrf2 is also involved in infections caused by other protozoan microorganisms like *Cryptosporidium parvum*, *Entamoeba histolytica*, *Toxoplasma gondii,* and *Plasmodium* spp. All of these infections result in the controlling of Nrf2 and a decreased anti-inflammatory and antioxidant profile [171,172,173,174]. For instance, *Trypanosoma cruzi*, which infects THP-1 cells, needs oxidative stress to successfully establish a parasitic relationship, while the overexpression of Nrf2 decreases infection [175]. Generally, very low oxidative stress is caused by *Leishmania amazonensis* infection [176]. Infection with *Leishmania amazonensis* results in the release of lower amounts of ROS, such as hydrogen peroxide, in comparison with *Leishmania major* (around 20 times lower). However, there is no information on the amount of Nrf2 activation during *L. major* infection, and the lower ROS generation levels from *L. amazonensis* infection may indicate the activation of the Nrf2 pathway and, as a result, a decrease in cellular oxidative stress [177]. 

After receiving IFN-I therapy, the primary establishment and multiplication of *L. amazonensis* infection is partially related to the enzyme superoxide dismutase 1 (SOD1), which is located downstream of Nrf2 and decreases the oxidative stress that is adverse to Leishmania and could boost the proliferation and affect the results of leishmaniasis [178,179]. Patients with cutaneous leishmaniasis infected by *L. braziliensis* or *L. amazonensis* have higher levels of SOD1, which might be employed as biomarkers of these diseases [180]. Additionally, there is a positive correlation between parasite SOD2/4 levels and host SOD1 levels, indicating that the connection between the parasite and host controls the expression of both genomes’ genes. Changes in the serum component levels of patients with cutaneous leishmaniasis have previously been noted, which may indicate that antioxidant enzyme cofactors may be involved in the infection [181]. Research has shown that *L. amazonensis* infection stimulates dsRNA-induced kinase (PKR), which aids in the parasite’s proliferation in macrophages [176,182,183,184,185,186]. This proposes that *L. amazonensis* has evolved the ability to take advantage of this signaling pathway in the host cell for its own gain. Because PKR is a signal transduction protein, it is reasonable to assume that there will be a rise in the production of inflammatory mediators that are related to this kinase. As an illustration, certain cytokines (IL-10 and IL-27) and antioxidative enzymes (SOD1 and HO-1) encourage the establishment of infection and create an intracellular environment for the development of leishmaniasis. Infection with *L. amazonensis* increases SOD1 expression in mouse peritoneal and human macrophage lineages in a PKR/Nrf2-dependent way [176]. Additionally, these species use the PI3K/Akt pathway to cause Nrf2 to translocate into the nucleus and bind to ARE in the promoters of p62, SOD1, and Nrf2. Also, ROS, peroxynitrite, and nitric oxide levels are reduced in infected macrophages, and amastigotes replicate less often in Nrf2-deficient cells than in wild-type cells. Infections generated by *L. braziliensis* and certain strains of *L. amazonensis* (from patients with localized or widespread cutaneous leishmaniasis), in addition to those caused by *L. amazonensis*, also firmly modify the PKR/Nrf2 axis.

Numerous researchers have provided evidence that oxidative stress and the autophagic process are related [187]. Autophagy stimulation decreases NO burst generation in *L. major* and *L. amazonensis* infections without altering in the activity of arginase [188]. Due to the ability of *L. amazonensis* infection to trigger autophagy, which is necessary for infection development [189], the involvement of the PI3K, PKR, and Nrf2 pathways in this cellular process have been documented. These signaling pathways are closely related or convergent, and as a result, they may work synergistically to cause autophagy and the development of an antioxidative profile during *L. amazonensis* infection. Furthermore, because PKR phosphorylating eIF2α is essential for controlling autophagosomes, PKR-deficient cells exhibit decreased autophagic activities [190]. Keap1 levels are decreased in *L. amazonensis*-infected macrophages, allowing Nrf2 to enter the nucleus and alter the expression of ARE-dependent genes. The involvement of the ARE-, pI3K-, IFN-1/PKR-, and autophagy-signaling pathways in the infection is supported by in situ transcriptomics studies using samples from individuals who had an *L. braziliensis* infection [176]. Additionally, it was shown that *L. amazonensis* infection lowered Nrf2 expression and nuclear translocation, decreased HO-1 (heme oxygenase-1) expression, and enhanced nitric oxide generation in ATF4 (activating transcription factor 4)-deficient macrophages [191]. In the same study, it was shown that PERK phosphorylation induced by endoplasmic reticulum stress resulted in the activation of Nrf2, which caused the dimerization of ATF4 in the nucleus and boosted the Nrf2/ATF4 regulation of ARE in the HO-1 gene promoter, preferring *L. amazonensis* infection. HO-1 is a crucial enzyme that is activated by cellular stress. It possesses anti-inflammatory and antioxidant characteristics as well as a catalytic role that promotes Leishmania infection [192]. A phlebotomine-type sandfly, *Lutzomyia longipalpis* Saliva, causes Nrf2 production and activates the HO-1 target gene in macrophages and human skin at the site of a bite, illuminating the mechanism by which sandfly-borne vectors spread and develop Leishmania infections [193]. However, despite likenesses in Leishmania species, depending on the immunological profile of the host, the species exhibit various patterns of virulence and diseases. According to the proteomics comparison analysis of *L. major* and *L. amazonensis* infections, the canonical signature of the Nrf2 pathway is outstanding in *L. amazonensis*-infected macrophages, as demonstrated by a remarkable increase in the expression of HO-1 and SQSTM1 (p62), indicating that this pathway is not used by *L. major* to undermine the defences of the host cell [194]. Macrophages infected with *L. donovani* take advantage of Nrf2 activation as well, while the parasite uses a specific pathway to survive in macrophages through increasing Tollip (toll-interacting protein) expression, a negative regulator of the activation of the IL-1R/TLR pathway [195].

### 3.4. Nrf2 and Viral Infection

It has been challenging to distinguish whether indirect Nrf2 activation during viral infection is the virus’ desired outcome or merely the result of the host’s defensive response to infection. Numerous studies show direct or indirect mechanisms that activate Nrf2 after infection with a wide range of viruses. Nrf2 activation by the Marburgvirus is one of the best examples of direct Nrf2 induction by a virus [196]. According to Page et al., mice with the Nrf2 allele deleted are more resistant to Marburgvirus infection. It has been demonstrated that VP24, a Marburgvirus viral protein, can bind to Keap1 and release Nrf2 while inhibiting the ubiquitin ligase activity of the Keap1-Cul3-Rbx1 complex, therefore activating Nrf2 [196,197]. Kosmider et al. demonstrated that 48 h after Influenza A virus (IAV) infection, ROS generation is increased, Nrf2 is activated, and the downstream effector of Nrf2, HO-1, is upregulated at both the mRNA and protein levels. The findings imply that IVA infection triggers Nrf2 activation, probably by triggering ROS [198]. Olagnier et al. also reported that the expression of Nrf2’s downstream effectors, including heme oxygenase-1 (HMOX-1), superoxide dismutase 2 (SOD2), NQO1, glutamate-cysteine ligase catalytic subunit (GCLC), and GCLM, is subsequently induced by dengue virus (DENV) infection in initial differentiated dendritic cells [199]. In another study, Sun et al. found that the respiratory syncytial virus (RSV) infects a human alveolar basal epithelial cell line (A549) in vitro, leading to Nrf2 induction [200]. They showed an increase in Nrf2 after 24 h of infection. They indicated that the activation mechanism of Nrf2 is unknown, but Nrf2 activation leads to the upregulation of TLR7 expression. In a similar study, Komaravelli et al. also reported on Nrf2 activation in response to RSV infection [201]. They reported the activation of Nrf2 in the early hours of infection, while Sun et al. reported Nrf2 activation in the late hours of infection. Mastrantonio et al. indicated that Tat, a transcription factor of the human immunodeficiency virus (HIV), stimulates Nrf2 in neuroblastoma cell lines via raising ROS levels. They demonstrated that the virus did not directly infect the neuroblastoma cells in their study; the activation of Nrf2 was caused by an increase in ROS generated by HIV-infected macrophages or glia cells in a Tat-dependent way. Therefore, Nrf2 was activated in this situation as a result of an infection-related bystander response [202]. The result of the study by Liu et al. showed that the human hepatitis B virus (HBV) in hepatocyte cell lines and human liver tissue could activate Nrf2 production [203]. Ivanov et al. also found that the Nrf2/ARE pathway was activated by the hepatitis C virus (HCV) in a human liver cell line [204]. They reported that the activation of the Nrf2/ARE pathway is mediated by several independent mechanisms through the core proteins of HCV. Lee et al. reported an increase in Nrf2 in primary human foreskin fibroblasts (HFFs) when they were infected by human cytomegalovirus (HCMV) [205]. They found that the level of Nrf2 was increased in the nucleus 24 h post-infection. Choi et al. reported that non-structural proteins (NSs) of severe fever with thrombocytopenia syndrome virus (SFTSV) prevent the function of tripartite motif 21 (TRIM21) to upregulate the p62-Keap1-Nrf2 antioxidant pathway for efficient viral pathogenesis [206].

### 3.5. Nrf2 and Bacterial Infection

It has been shown that bacterial infection can also cause Nrf2 activation. Nairz et al. demonstrated that *Salmonella typhimurium* infection activates Nrf2 in macrophages [207]. They found that nitric oxide synthase 2 activates Nrf2, resulting in increased Fpn1 transcription, cellular iron export, and the limitation of iron availability for pathogens, thereby preventing pathogen proliferation. Gomez et al. reported that *Streptococcus pneumoniae* infection results in an increased early host defence in Nrf2-deficient mice, which reduces neutrophil recruitment. They demonstrated that when inoculum numbers increased, mortality in Nrf2-null mice increased dramatically from 15% to 31% and 100%, while all wild-type (WT) mice survived and Nrf2-null mice had a failure in clearance, especially at the middle dosage [208]. In another study, Joshi et al. found that the Nrf2 pathway is activated in response to uropathogenic *Escherichia coli* (UPEC)-triggered ROS production [209]. They showed the molecular process by which the activation of Nrf2 in urothelial cells decreases the generation of ROS, inflammation, and cell death, increases the evacuation of UPEC, and lowers the bacterial burden. On the other hand, the [191] deletion of Nrf2 increases bacterial load, inflammation, and ROS generation both in vitro and in vivo. In a different study, Harvey et al. investigated whether sulforaphane, a phytochemical, activated Nrf2 and restored alveolar macrophages’ ability to phagocytose nontypeable *Haemophilus influenzae* (NTHI) and *Pseudomonas aeruginosa* (PA) in patients with chronic obstructive pulmonary disease (COPD) [210]. They demonstrated that Nrf2 enhanced macrophages’ capacity to phagocytose by directly upregulating the transcription of the scavenger receptor MARCO. They also reported that Nrf2 or MARCO alteration prevented COPD alveolar macrophages from phagocytosing bacteria in a sulforaphane-mediated manner [210]. Their findings support the use of pharmaceutical medicines like sulforaphane to target this pathway and prevent COPD exacerbations caused by bacterial infection by highlighting the significance of Nrf2 and its downstream target MARCO in enhancing antibacterial defences. The role of Nrf2 in a pathogenic setting is summarized in Table 2. 

### 3.6. Nrf2 and Resistance to Infection

Tissue damage as well as disease tolerance to infections can be controlled by Nrf2 signaling. 

One of the mechanisms is the formation of functional crosstalk between the gasotransmitters NO and CO, as demonstrated for *Plasmodium* infection [211,212]. It has been shown that both CO [211,213,214] and NO [212,215,216] can repress the development of experimental cerebral malaria in mice [217]. This defensive effect acts via the activation of Nrf2 through NO [218], probably through a mechanism that targets Keap1 at Cys151 [24,219]; however, this has not been shown experimentally. The production of CO and the expression of HO-1 is induced by the activation of Nrf2 through the catabolism of heme by HO-1, which finally inhibits the progress of experimental cerebral malaria [218]. This happens through a mechanism that involves the joining of CO to the prosthetic heme group of cell-free haemoglobin produced during the blood stage of *Plasmodium* infection. Therefore, it prevents heme from taking apart in the pathogenesis of experimental cerebral malaria [211,213,214,218]. It has been proposed that the interaction between two gasotransmitters provides disease tolerance to Plasmodium infection via a mechanism controlled by Nrf2 due to the fact that the defensive effect used by the NO > Nrf2 > HO-1 > CO signal transduction pathway does not correspond with the modulation of the host pathogen load [218,220]. Nrf2 likely regulates heme/iron metabolism through the production of Nrf2-responsive genes, which in turn regulate tissue damage and disease resistance to malaria [221]. These involve the iron storage protein ferritin H chain (FtH) [222,223], which can protect tissues against damage and cause disease tolerance to malaria in mice. In another study, Ferreira et al. showed that activating the Nrf2 signal transduction pathway can protect transgenic sickle hemoglobin mice from cerebral malaria [213,214]. It was reported that disease tolerance to Plasmodium infection can be caused by sickle hemoglobin [213,220]. Nrf2 regulates a mechanism by which HO-1 expression can be induced by sickle hemoglobin, resulting in CO production and, therefore, the control of tissue damage and disease tolerance to malaria [214]. Although more studies are needed to investigate the mechanisms behind human protection from malaria by sickle hemoglobin, it is likely that the above-mentioned mechanism is the reason. A similar mechanism by other chronic hemolytic conditions like hemoglobin C [224,224], glucose 6 phosphate dehydrogenase (G6PD) deficiency in males [225], β- or α-thalassemia [226], and mutations underlying red blood cell cytoskeleton or membrane protein defects may also exert a protective effect against malaria [227]. The various mechanisms that converge at the activation level of Nrf2 likely underlie the protective effect, which is linked to these mutations. It is therefore conceivable that protection against malaria due to a limitation in the severity of disease caused by these mutations is because of the co-evolution of these mutations with the Nrf2 signal transduction pathway, as shown forsickle hemoglobin [213].

## 4. Conclusions

In this concise review, we summarized the roles Nrf2 in DNA damage and repair, stem cell self-renewal, differentiation, and cellular senescence as well as its critical role in infections. Furthermore, the Nrf2 pathway acts as a driver of cancer progression and is resistant to radiotherapy as well as chemotherapy, but still the oncogenic role of Nrf2 is controversial due to its contribution in overcoming chemical carcinogenesis. Yet, the achievement of an optimal balance between stem cell fate control and the suppression of tumorigenesis is highly required for promising therapy based on the modification of the Nrf2 level. Pathogens are not negatively impacted by disease tolerance mechanisms. As a result, the disease can spread from the infected host even when they are healthy due to stress reactions that underlie disease tolerance. The natural selection of genes controlling stress responses, such as Nrf2, is likely to be significantly impacted by this [228]. Furthermore, stress responses protect essential cellular processes at the expense of ‘accessory’ ones [229,230,231], necessitating their strict regulation over time [220]. The finding that chronic Nrf2 activation increases tumorigenesis serves as an example that Nrf2 is not an exception to this rule [30]. Mechanisms through the process of evolution have caused the development of an anti-oxidative pathway, which is supported by Nrf2 signaling, that works to reduce oxidative burst in the host cell and infections. Though the host cell is partially ready to respond to infections that disrupt its homeostasis, pathogens attempt to disrupt host cell signaling and modify critical mediators for the formation and spread of the infection. The stress response controlled by Nrf2 is likely to have a key role in giving diseases tolerance to systemic infections, like bacterial and *Plasmodium* infections. The initial mechanism in the development of infections is probably co-evolution, which has drawn a great deal of interest in the study of these mechanisms.

Finally, additional studies are required to identify the key sensors involved in Nrf2 activation by various pathogen species. The possibilities of identifying potential therapeutic targets will become more apparent with the discovery of Nrf2’s molecular partners and the variety of genes that may be implicated in the oxidative burst.

## Figures and Tables

**Table 2:** Nrf2 in pathogenic setting.

Mediator	Role	Impact	Reference
*Trypanosoma cruzi*	Nrf2 activation	Reduces parasitism	[175]
*L. amazonensis*	Increases superoxide dismutase 1 (SOD1)	Activates Nrf2 and, therefore, decreases the level of ROS, nitric oxide, and peroxynitrite in infected macrophages	[176]
*L. amazonensis*	Reduces Keap1 levels	Enables the translocation of Nrf2 into the nucleus and modulate ARE-dependent gene expression	[189]
*L. amazonensis*	Decreases Nrf2 expression	Increases nitric oxide production	[191]
VP24, a viral protein of Marburgvirus	Binds to Keap1	Induces the release of Nrf2 and inhibits the ubiquitin ligase activity of the Keap1–Cul3–Rbx1 complex	[196,197]
Influenza A Virus (IAV)	Increases ROS production	Induces the activation of Nrf2	[198]
Dengue virus		Nrf2 activation	[199]
Respiratory syncytial virus (RSV)	Unknown	Induces Nrf2	[200,201]
Human immunodeficiency virus (HIV)	Increases ROS	Nrf2 activation	[202]
Human hepatitis B virus (HBV)	Nrf2 activation	Nrf2 activation	[203]
Hepatitis C virus (HCV)	Core proteins of HCV	Nrf2/ARE activation	[204]
Human cytomegalovirus (HCMV)	Increases Nrf2 expression	Increases Nrf2 in primary human foreskin fibroblasts	[205]
Non-structural proteins (NSs) of severe fever with thrombocytopenia syndrome virus (SFTSV)	Inhibits the function of tripartite motif 21 (TRIM21)	Inhibits the upregulation of the p62-Keap1-Nrf2 antioxidant pathway	[206]
*Salmonella typhimurium*	Nrf2 activation	Increases resistance to infection	[207]
*Streptococcus pneumoniae*	Nrf2 activation	Increases resistance to infection	[208]
Uropathogenic *Escherichia coli* (UPEC)	Nrf2 activation	Reduces ROS production and bacterial load	[209]
Nontypeable *Hemophilus influenzae* (NTHI) and *Pseudomonas aeruginosa* (PA)	Nrf2 activation	Increases the phagocytic ability of macrophages	[210]

## Data Availability

Data is contained within the article.

## References

[1] Gutteridge J.M., Halliwell B. (1992). Comments on review of Free Radicals in Biology and Medicine, second edition, by Barry Halliwell and John M. C. Gutteridge. Free Radic. Biol. Med..

[2] Sies H., Jones D.P. (2020). Reactive oxygen species (ROS) as pleiotropic physiological signaling agents. Nat. Rev. Mol. Cell Biol..

[3] Bhattacharyya A., Chattopadhyay R., Mitra S., Crowe S.E. (2014). Oxidative stress: An essential factor in the pathogenesis of gastrointestinal mucosal diseases. Physiol. Rev..

[4] Hawkins C.L., Davies M.J. (2019). Detection, identification, and quantification of oxidative protein modifications. J. Biol. Chem..

[5] Carocho M., Ferreira I.C. (2013). A review on antioxidants, prooxidants and related controversy: Natural and synthetic compounds, screening and analysis methodologies and future perspectives. Food Chem. Toxicol..

[6] Neha K., Haider M.R., Pathak A., Yar M.S. (2019). Medicinal prospects of antioxidants: A review. Eur. J. Med. Chem..

[7] Turell L., Zeida A., Trujillo M. (2020). Mechanisms and consequences of protein cysteine oxidation: The role of the initial short-lived intermediates. Essays Biochem..

[8] Garrido Ruiz D., Sandoval-Perez A., Rangarajan A.V., Gunderson E.L., Jacobson M.P. (2022). Cysteine Oxidation in Proteins: Structure, Biophysics, and Simulation. Biochemistry.

[9] Timme-Laragy A.R., Hahn M.E., Hansen J.M., Rastogi A., Roy M.A. (2018). Redox stress and signaling during vertebrate embryonic development: Regulation and responses. Semin. Cell Dev. Biol..

[10] Oswald M.C.W., Garnham N., Sweeney S.T., Landgraf M. (2018). Regulation of neuronal development and function by ROS. FEBS Lett..

[11] Dorrington M.G., Fraser I.D.C. (2019). NF-kappaB Signaling in Macrophages: Dynamics, Crosstalk, and Signal Integration. Front. Immunol..

[12] Ernst O., Vayttaden S.J., Fraser I.D.C. (2018). Measurement of NF-kappaB Activation in TLR-Activated Macrophages. Methods Mol. Biol..

[13] Lemmens K.J., Herst P.M., Housmans B.A., Moalin M., van der Vijgh W.J., Bast A., Haenen G.R. (2015). The contribution of the major metabolite 4′-O-methylmonoHER to the antioxidant activity of the flavonoid monoHER. Chem. Biol. Interact..

[14] Chen N., Wu L., Yuan H., Wang J. (2015). ROS/Autophagy/Nrf2 Pathway Mediated Low-Dose Radiation Induced Radio-Resistance in Human Lung Adenocarcinoma A549 Cell. Int. J. Biol. Sci..

[15] Pizzino G., Irrera N., Cucinotta M., Pallio G., Mannino F., Arcoraci V., Squadrito F., Altavilla D., Bitto A. (2017). Oxidative Stress: Harms and Benefits for Human Health. Oxid. Med. Cell. Longev..

[16] Davalli P., Mitic T., Caporali A., Lauriola A., D’Arca D. (2016). ROS, Cell Senescence, and Novel Molecular Mechanisms in Aging and Age-Related Diseases. Oxid. Med. Cell. Longev..

[17] Villalpando-Rodriguez G.E., Gibson S.B. (2021). Reactive Oxygen Species (ROS) Regulates Different Types of Cell Death by Acting as a Rheostat. Oxid. Med. Cell. Longev..

[18] Pastor R., Tur J.A. (2019). Antioxidant Supplementation and Adaptive Response to Training: A Systematic Review. Curr. Pharm. Des..

[19] Tonelli C., Chio I.I.C., Tuveson D.A. (2018). Transcriptional Regulation by Nrf2. Antioxid. Redox Signal..

[20] Moi P., Chan K., Asunis I., Cao A., Kan Y.W. (1994). Isolation of NF-E2-related factor 2 (Nrf2), a NF-E2-like basic leucine zipper transcriptional activator that binds to the tandem NF-E2/AP1 repeat of the beta-globin locus control region. Proc. Natl. Acad. Sci. USA.

[21] Vinson C., Myakishev M., Acharya A., Mir A.A., Moll J.R., Bonovich M. (2002). Classification of human B-ZIP proteins based on dimerization properties. Mol. Cell. Biol..

[22] Motohashi H., Shavit J.A., Igarashi K., Yamamoto M., Engel J.D. (1997). The world according to Maf. Nucleic Acids Res..

[23] Dodson M., de la Vega M.R., Cholanians A.B., Schmidlin C.J., Chapman E., Zhang D.D. (2019). Modulating NRF2 in Disease: Timing Is Everything. Annu. Rev. Pharmacol. Toxicol..

[24] Hayes J.D., Dinkova-Kostova A.T. (2014). The Nrf2 regulatory network provides an interface between redox and intermediary metabolism. Trends Biochem. Sci..

[25] Goshtasbi H., Pakchin P.S., Movafeghi A., Barar J., Castejon A.M., Omidian H., Omidi Y. (2022). Impacts of oxidants and antioxidants on the emergence and progression of Alzheimer’s disease. Neurochem. Int..

[26] Stewart D., Killeen E., Naquin R., Alam S., Alam J. (2003). Degradation of transcription factor Nrf2 via the ubiquitin-proteasome pathway and stabilization by cadmium. J. Biol. Chem..

[27] Torrente L., DeNicola G.M. (2022). Targeting NRF2 and Its Downstream Processes: Opportunities and Challenges. Annu. Rev. Pharmacol. Toxicol..

[28] Szanto A., Narkar V., Shen Q., Uray I.P., Davies P.J., Nagy L. (2004). Retinoid X receptors: X-ploring their (patho)physiological functions. Cell Death Differ..

[29] Katsuoka F., Yamamoto M. (2016). Small Maf proteins (MafF, MafG, MafK): History, structure and function. Gene.

[30] DeNicola G.M., Karreth F.A., Humpton T.J., Gopinathan A., Wei C., Frese K., Mangal D., Yu K.H., Yeo C.J., Calhoun E.S. (2011). Oncogene-induced Nrf2 transcription promotes ROS detoxification and tumorigenesis. Nature.

[31] Li W., Thakor N., Xu E.Y., Huang Y., Chen C., Yu R., Holcik M., Kong A.N. (2010). An internal ribosomal entry site mediates redox-sensitive translation of Nrf2. Nucleic Acids Res..

[32] Sun Z., Huang Z., Zhang D.D. (2009). Phosphorylation of Nrf2 at multiple sites by MAP kinases has a limited contribution in modulating the Nrf2-dependent antioxidant response. PLoS ONE.

[33] Karihtala P., Porvari K., Soini Y., Haapasaari K.M. (2017). Redox Regulating Enzymes and Connected MicroRNA Regulators Have Prognostic Value in Classical Hodgkin Lymphomas. Oxid. Med. Cell. Longev..

[34] Sangokoya C., Telen M.J., Chi J.T. (2010). microRNA miR-144 modulates oxidative stress tolerance and associates with anemia severity in sickle cell disease. Blood.

[35] Yang M., Yao Y., Eades G., Zhang Y., Zhou Q. (2011). MiR-28 regulates Nrf2 expression through a Keap1-independent mechanism. Breast Cancer Res. Treat..

[36] Cuadrado A. (2015). Structural and functional characterization of Nrf2 degradation by glycogen synthase kinase 3/beta-TrCP. Free Radic. Biol. Med..

[37] Nam L.B., Keum Y.S. (2019). Binding partners of NRF2: Functions and regulatory mechanisms. Arch. Biochem. Biophys..

[38] Zhang D.D., Chapman E. (2020). The role of natural products in revealing NRF2 function. Nat. Prod. Rep..

[39] Otsuki A., Yamamoto M. (2020). Cis-element architecture of Nrf2-sMaf heterodimer binding sites and its relation to diseases. Arch. Pharm. Res..

[40] Igarashi K., Kataoka K., Itoh K., Hayashi N., Nishizawa M., Yamamoto M. (1994). Regulation of transcription by dimerization of erythroid factor NF-E2 p45 with small Maf proteins. Nature.

[41] Itoh K., Chiba T., Takahashi S., Ishii T., Igarashi K., Katoh Y., Oyake T., Hayashi N., Satoh K., Hatayama I. (1997). An Nrf2/small Maf heterodimer mediates the induction of phase II detoxifying enzyme genes through antioxidant response elements. Biochem. Biophys. Res. Commun..

[42] Toki T., Itoh J., Kitazawa J., Arai K., Hatakeyama K., Akasaka J., Igarashi K., Nomura N., Yokoyama M., Yamamoto M. (1997). Human small Maf proteins form heterodimers with CNC family transcription factors and recognize the NF-E2 motif. Oncogene.

[43] Ellenberger T., Fass D., Arnaud M., Harrison S.C. (1994). Crystal structure of transcription factor E47: E-box recognition by a basic region helix-loop-helix dimer. Genes Dev..

[44] Raghunath A., Sundarraj K., Nagarajan R., Arfuso F., Bian J., Kumar A.P., Sethi G., Perumal E. (2018). Antioxidant response elements: Discovery, classes, regulation and potential applications. Redox Biol..

[45] Kataoka K., Noda M., Nishizawa M. (1994). Maf nuclear oncoprotein recognizes sequences related to an AP-1 site and forms heterodimers with both Fos and Jun. Mol. Cell. Biol..

[46] Kurokawa H., Motohashi H., Sueno S., Kimura M., Takagawa H., Kanno Y., Yamamoto M., Tanaka T. (2009). Structural basis of alternative DNA recognition by Maf transcription factors. Mol. Cell. Biol..

[47] Itoh K., Wakabayashi N., Katoh Y., Ishii T., Igarashi K., Engel J.D., Yamamoto M. (1999). Keap1 represses nuclear activation of antioxidant responsive elements by Nrf2 through binding to the amino-terminal Neh2 domain. Genes Dev..

[48] Kang M.I., Kobayashi A., Wakabayashi N., Kim S.G., Yamamoto M. (2004). Scaffolding of Keap1 to the actin cytoskeleton controls the function of Nrf2 as key regulator of cytoprotective phase 2 genes. Proc. Natl. Acad. Sci. USA.

[49] Zipper L.M., Mulcahy R.T. (2002). The Keap1 BTB/POZ dimerization function is required to sequester Nrf2 in cytoplasm. J. Biol. Chem..

[50] Zhang D.D., Lo S.C., Cross J.V., Templeton D.J., Hannink M. (2004). Keap1 is a redox-regulated substrate adaptor protein for a Cul3-dependent ubiquitin ligase complex. Mol. Cell. Biol..

[51] Tong K.I., Katoh Y., Kusunoki H., Itoh K., Tanaka T., Yamamoto M. (2006). Keap1 recruits Neh2 through binding to ETGE and DLG motifs: Characterization of the two-site molecular recognition model. Mol. Cell. Biol..

[52] Lo S.C., Li X., Henzl M.T., Beamer L.J., Hannink M. (2006). Structure of the Keap1:Nrf2 interface provides mechanistic insight into Nrf2 signaling. EMBO J..

[53] Dayalan Naidu S., Dinkova-Kostova A.T. (2020). KEAP1, a cysteine-based sensor and a drug target for the prevention and treatment of chronic disease. Open Biol..

[54] Nishida M., Yamashita N., Ogawa T., Koseki K., Warabi E., Ohue T., Komatsu M., Matsushita H., Kakimi K., Kawakami E. (2021). Mitochondrial reactive oxygen species trigger metformin-dependent antitumor immunity via activation of Nrf2/mTORC1/p62 axis in tumor-infiltrating CD8T lymphocytes. J. Immunother. Cancer.

[55] Dondelinger Y., Jouan-Lanhouet S., Divert T., Theatre E., Bertin J., Gough P.J., Giansanti P., Heck A.J., Dejardin E., Vandenabeele P. (2015). NF-kappaB-Independent Role of IKKalpha/IKKbeta in Preventing RIPK1 Kinase-Dependent Apoptotic and Necroptotic Cell Death during TNF Signaling. Mol. Cell.

[56] Emanuele S., Lauricella M., D’Anneo A., Carlisi D., De Blasio A., Di Liberto D., Giuliano M. (2020). p62: Friend or Foe? Evidences for OncoJanus and NeuroJanus Roles. Int. J. Mol. Sci..

[57] Wei R., Enaka M., Muragaki Y. (2019). Activation of KEAP1/NRF2/P62 signaling alleviates high phosphate-induced calcification of vascular smooth muscle cells by suppressing reactive oxygen species production. Sci. Rep..

[58] Nioi P., Nguyen T., Sherratt P.J., Pickett C.B. (2005). The carboxy-terminal Neh3 domain of Nrf2 is required for transcriptional activation. Mol. Cell. Biol..

[59] Katoh Y., Itoh K., Yoshida E., Miyagishi M., Fukamizu A., Yamamoto M. (2001). Two domains of Nrf2 cooperatively bind CBP, a CREB binding protein, and synergistically activate transcription. Genes Cells.

[60] Sekine H., Okazaki K., Ota N., Shima H., Katoh Y., Suzuki N., Igarashi K., Ito M., Motohashi H., Yamamoto M. (2016). The Mediator Subunit MED16 Transduces NRF2-Activating Signals into Antioxidant Gene Expression. Mol. Cell. Biol..

[61] Zhang J., Ohta T., Maruyama A., Hosoya T., Nishikawa K., Maher J.M., Shibahara S., Itoh K., Yamamoto M. (2006). BRG1 interacts with Nrf2 to selectively mediate HO-1 induction in response to oxidative stress. Mol. Cell. Biol..

[62] Kim J.H., Yu S., Chen J.D., Kong A.N. (2013). The nuclear cofactor RAC3/AIB1/SRC-3 enhances Nrf2 signaling by interacting with transactivation domains. Oncogene.

[63] Ki S.H., Cho I.J., Choi D.W., Kim S.G. (2005). Glucocorticoid receptor (GR)-associated SMRT binding to C/EBPbeta TAD and Nrf2 Neh4/5: Role of SMRT recruited to GR in GSTA2 gene repression. Mol. Cell. Biol..

[64] Alam M.M., Okazaki K., Nguyen L.T.T., Ota N., Kitamura H., Murakami S., Shima H., Igarashi K., Sekine H., Motohashi H. (2017). Glucocorticoid receptor signaling represses the antioxidant response by inhibiting histone acetylation mediated by the transcriptional activator NRF2. J. Biol. Chem..

[65] Wang H., Liu K., Geng M., Gao P., Wu X., Hai Y., Li Y., Li Y., Luo L., Hayes J.D. (2013). RXRalpha inhibits the NRF2-ARE signaling pathway through a direct interaction with the Neh7 domain of NRF2. Cancer Res..

[66] Chowdhry S., Zhang Y., McMahon M., Sutherland C., Cuadrado A., Hayes J.D. (2013). Nrf2 is controlled by two distinct beta-TrCP recognition motifs in its Neh6 domain, one of which can be modulated by GSK-3 activity. Oncogene.

[67] Hughes K., Nikolakaki E., Plyte S.E., Totty N.F., Woodgett J.R. (1993). Modulation of the glycogen synthase kinase-3 family by tyrosine phosphorylation. EMBO J..

[68] Cross D.A., Alessi D.R., Cohen P., Andjelkovich M., Hemmings B.A. (1995). Inhibition of glycogen synthase kinase-3 by insulin mediated by protein kinase B. Nature.

[69] Fang X., Yu S.X., Lu Y., Bast R.C., Woodgett J.R., Mills G.B. (2000). Phosphorylation and inactivation of glycogen synthase kinase 3 by protein kinase A. Proc. Natl. Acad. Sci. USA.

[70] Fang X., Yu S., Tanyi J.L., Lu Y., Woodgett J.R., Mills G.B. (2002). Convergence of multiple signaling cascades at glycogen synthase kinase 3: Edg receptor-mediated phosphorylation and inactivation by lysophosphatidic acid through a protein kinase C-dependent intracellular pathway. Mol. Cell. Biol..

[71] Goode N., Hughes K., Woodgett J.R., Parker P.J. (1992). Differential regulation of glycogen synthase kinase-3 beta by protein kinase C isotypes. J. Biol. Chem..

[72] Bellezza I., Giambanco I., Minelli A., Donato R. (2018). Nrf2-Keap1 signaling in oxidative and reductive stress. Biochim. Biophys. Acta Mol. Cell Res..

[73] Joo M.S., Kim W.D., Lee K.Y., Kim J.H., Koo J.H., Kim S.G. (2016). AMPK Facilitates Nuclear Accumulation of Nrf2 by Phosphorylating at Serine 550. Mol. Cell. Biol..

[74] Jain A.K., Jaiswal A.K. (2007). GSK-3beta acts upstream of Fyn kinase in regulation of nuclear export and degradation of NF-E2 related factor 2. J. Biol. Chem..

[75] Plafker K.S., Nguyen L., Barneche M., Mirza S., Crawford D., Plafker S.M. (2010). The ubiquitin-conjugating enzyme UbcM2 can regulate the stability and activity of the antioxidant transcription factor Nrf2. J. Biol. Chem..

[76] Singh B., Chatterjee A., Ronghe A.M., Bhat N.K., Bhat H.K. (2013). Antioxidant-mediated up-regulation of OGG1 via NRF2 induction is associated with inhibition of oxidative DNA damage in estrogen-induced breast cancer. BMC Cancer.

[77] Kim S.B., Pandita R.K., Eskiocak U., Ly P., Kaisani A., Kumar R., Cornelius C., Wright W.E., Pandita T.K., Shay J.W. (2012). Targeting of Nrf2 induces DNA damage signaling and protects colonic epithelial cells from ionizing radiation. Proc. Natl. Acad. Sci. USA.

[78] Jayakumar S., Pal D., Sandur S.K. (2015). Nrf2 facilitates repair of radiation induced DNA damage through homologous recombination repair pathway in a ROS independent manner in cancer cells. Mutat. Res..

[79] McDonald J.T., Kim K., Norris A.J., Vlashi E., Phillips T.M., Lagadec C., Della Donna L., Ratikan J., Szelag H., Hlatky L. (2010). Ionizing radiation activates the Nrf2 antioxidant response. Cancer Res..

[80] Sharma V., Collins L.B., Chen T.H., Herr N., Takeda S., Sun W., Swenberg J.A., Nakamura J. (2016). Oxidative stress at low levels can induce clustered DNA lesions leading to NHEJ mediated mutations. Oncotarget.

[81] Dianov G.L., Hubscher U. (2013). Mammalian base excision repair: The forgotten archangel. Nucleic Acids Res..

[82] Ciccia A., Elledge S.J. (2010). The DNA damage response: Making it safe to play with knives. Mol. Cell.

[83] Davies A.A., Masson J.Y., McIlwraith M.J., Stasiak A.Z., Stasiak A., Venkitaraman A.R., West S.C. (2001). Role of BRCA2 in control of the RAD51 recombination and DNA repair protein. Mol. Cell.

[84] Rotman G., Shiloh Y. (1998). ATM: From gene to function. Hum. Mol. Genet..

[85] Khalil H.S., Tummala H., Hupp T.R., Zhelev N. (2012). Pharmacological inhibition of ATM by KU55933 stimulates ATM transcription. Exp. Biol. Med..

[86] Paull T.T., Rogakou E.P., Yamazaki V., Kirchgessner C.U., Gellert M., Bonner W.M. (2000). A critical role for histone H2AX in recruitment of repair factors to nuclear foci after DNA damage. Curr. Biol..

[87] Khanna K.K., Jackson S.P. (2001). DNA double-strand breaks: Signaling, repair and the cancer connection. Nat. Genet..

[88] Zou L., Elledge S.J. (2003). Sensing DNA damage through ATRIP recognition of RPA-ssDNA complexes. Science.

[89] Cuadrado M., Martinez-Pastor B., Murga M., Toledo L.I., Gutierrez-Martinez P., Lopez E., Fernandez-Capetillo O. (2006). ATM regulates ATR chromatin loading in response to DNA double-strand breaks. J. Exp. Med..

[90] Adams K.E., Medhurst A.L., Dart D.A., Lakin N.D. (2006). Recruitment of ATR to sites of ionising radiation-induced DNA damage requires ATM and components of the MRN protein complex. Oncogene.

[91] Smits V.A., Reaper P.M., Jackson S.P. (2006). Rapid PIKK-dependent release of Chk1 from chromatin promotes the DNA-damage checkpoint response. Curr. Biol..

[92] Boutros R., Dozier C., Ducommun B. (2006). The when and wheres of CDC25 phosphatases. Curr. Opin. Cell Biol..

[93] Sekhar K.R., Freeman M.L. (2015). Nrf2 promotes survival following exposure to ionizing radiation. Free Radic. Biol. Med..

[94] Singh B., Bhat N.K., Bhat H.K. (2012). Induction of NAD(P)H-quinone oxidoreductase 1 by antioxidants in female ACI rats is associated with decrease in oxidative DNA damage and inhibition of estrogen-induced breast cancer. Carcinogenesis.

[95] Haghdoost S., Czene S., Naslund I., Skog S., Harms-Ringdahl M. (2005). Extracellular 8-oxo-dG as a sensitive parameter for oxidative stress in vivo and in vitro. Free Radic. Res..

[96] Haghdoost S., Sjolander L., Czene S., Harms-Ringdahl M. (2006). The nucleotide pool is a significant target for oxidative stress. Free Radic. Biol. Med..

[97] Lin S., Hao G., Long M., Lai F., Li Q., Xiong Y., Tian Y., Lai D. (2017). Oyster (*Ostrea plicatula* Gmelin) polysaccharides intervention ameliorates cyclophosphamide—Induced genotoxicity and hepatotoxicity in mice via the Nrf2-ARE pathway. Biomed. Pharmacother..

[98] Darband S.G., Sadighparvar S., Yousefi B., Kaviani M., Ghaderi-Pakdel F., Mihanfar A., Rahimi Y., Mobaraki K., Majidinia M. (2020). Quercetin attenuated oxidative DNA damage through NRF2 signaling pathway in rats with DMH induced colon carcinogenesis. Life Sci..

[99] Ross D., Siegel D. (2017). Functions of NQO1 in Cellular Protection and CoQ(10) Metabolism and its Potential Role as a Redox Sensitive Molecular Switch. Front. Physiol..

[100] Zhang B., Zhao J., Li S., Zeng L., Chen Y., Fang J. (2015). Mangiferin activates the Nrf2-ARE pathway and reduces etoposide-induced DNA damage in human umbilical cord mononuclear blood cells. Pharm. Biol..

[101] Lefaki M., Papaevgeniou N., Tur J.A., Vorgias C.E., Sykiotis G.P., Chondrogianni N. (2020). The dietary triterpenoid 18alpha-Glycyrrhetinic acid protects from MMC-induced genotoxicity through the ERK/Nrf2 pathway. Redox Biol..

[102] Srivastava R., Bhattacharya S., Chakraborty A., Chattopadhyay A. (2015). Differential in vivo genotoxicity of arsenic trioxide in glutathione depleted mouse bone marrow cells: Expressions of Nrf2/Keap1/P62. Toxicol. Mech. Methods.

[103] Sun X., Wang Y., Ji K., Liu Y., Kong Y., Nie S., Li N., Hao J., Xie Y., Xu C. (2020). NRF2 preserves genomic integrity by facilitating ATR activation and G2 cell cycle arrest. Nucleic Acids Res..

[104] Khalil H.S., Deeni Y. (2015). NRF 2 inhibition causes repression of ATM and ATR expression leading to aberrant DNA Damage Response. BioDiscovery.

[105] Trachootham D., Alexandre J., Huang P. (2009). Targeting cancer cells by ROS-mediated mechanisms: A radical therapeutic approach?. Nat. Rev. Drug Discov..

[106] Martinez-Outschoorn U.E., Peiris-Pages M., Pestell R.G., Sotgia F., Lisanti M.P. (2017). Cancer metabolism: A therapeutic perspective. Nat. Rev. Clin. Oncol..

[107] Hayes J.D., McMahon M. (2009). NRF2 and KEAP1 mutations: Permanent activation of an adaptive response in cancer. Trends Biochem. Sci..

[108] Godoy P., Pour Khavari A., Rizzo M., Sakamoto-Hojo E.T., Haghdoost S. (2020). Targeting NRF2, Regulator of Antioxidant System, to Sensitize Glioblastoma Neurosphere Cells to Radiation-Induced Oxidative Stress. Oxid. Med. Cell. Longev..

[109] Li W., Sun Y. (2020). Nrf2 is required for suppressing osteoclast RANKL-induced differentiation in RAW 264.7 cells via inactivating cannabinoid receptor type 2 with AM630. Regen. Ther..

[110] Garcia de Herreros A., Dunach M. (2019). Intracellular Signals Activated by Canonical Wnt Ligands Independent of GSK3 Inhibition and beta-Catenin Stabilization. Cells.

[111] Patel J.J., Butters O.R., Arnett T.R. (2014). PPAR agonists stimulate adipogenesis at the expense of osteoblast differentiation while inhibiting osteoclast formation and activity. Cell Biochem. Funct..

[112] Pakvasa M., Alverdy A., Mostafa S., Wang E., Fu L., Li A., Oliveira L., Athiviraham A., Lee M.J., Wolf J.M. (2017). Neural EGF-like protein 1 (NELL-1): Signaling crosstalk in mesenchymal stem cells and applications in regenerative medicine. Genes Dis..

[113] Kohan K., Carvajal R., Gabler F., Vantman D., Romero C., Vega M. (2010). Role of the transcriptional factors FOXO1 and PPARG on gene expression of SLC2A4 in endometrial tissue from women with polycystic ovary syndrome. Reproduction.

[114] Lowe C.E., O’Rahilly S., Rochford J.J. (2011). Adipogenesis at a glance. J. Cell Sci..

[115] Xiong Y., Zhao B., Zhang W., Jia L., Zhang Y., Xu X. (2020). Curcumin promotes osteogenic differentiation of periodontal ligament stem cells through the PI3K/AKT/Nrf2 signaling pathway. Iran J. Basic Med. Sci..

[116] Xue P., Hu X., Chang E., Wang L., Chen M., Wu T.H., Lee D.J., Foster B.L., Tseng H.C., Ko C.C. (2021). Deficiency of optineurin enhances osteoclast differentiation by attenuating the NRF2-mediated antioxidant response. Exp. Mol. Med..

[117] Xi X., Zhao Y., Liu H., Li Z., Chen S., Liu D. (2021). Nrf2 activation is involved in osteogenic differentiation of periodontal ligament stem cells under cyclic mechanical stretch. Exp. Cell Res..

[118] Yoon D.S., Choi Y., Lee J.W. (2016). Cellular localization of NRF2 determines the self-renewal and osteogenic differentiation potential of human MSCs via the P53-SIRT1 axis. Cell Death Dis..

[119] Kim J.H., Singhal V., Biswal S., Thimmulappa R.K., DiGirolamo D.J. (2014). Nrf2 is required for normal postnatal bone acquisition in mice. Bone Res..

[120] Haider N., Larose L. (2020). Activation of the PDGFRalpha-Nrf2 pathway mediates impaired adipocyte differentiation in bone marrow mesenchymal stem cells lacking Nck1. Cell Commun. Signal..

[121] Yang J., Sung J., Kim Y., Jeong H.S., Lee J. (2017). Inhibitory Effects of Butein on Adipogenesis through Upregulation of the Nrf2/HO-1 Pathway in 3T3-L1 Adipocytes. Prev. Nutr. Food Sci..

[122] Sun X., Li X., Jia H., Wang H., Shui G., Qin Y., Shu X., Wang Y., Dong J., Liu G. (2020). Nuclear Factor E2-Related Factor 2 Mediates Oxidative Stress-Induced Lipid Accumulation in Adipocytes by Increasing Adipogenesis and Decreasing Lipolysis. Antioxid. Redox Signal..

[123] Hou Y., Xue P., Bai Y., Liu D., Woods C.G., Yarborough K., Fu J., Zhang Q., Sun G., Collins S. (2012). Nuclear factor erythroid-derived factor 2-related factor 2 regulates transcription of CCAAT/enhancer-binding protein beta during adipogenesis. Free Radic. Biol. Med..

[124] Karaskov E., Scott C., Zhang L., Teodoro T., Ravazzola M., Volchuk A. (2006). Chronic palmitate but not oleate exposure induces endoplasmic reticulum stress, which may contribute to INS-1 pancreatic beta-cell apoptosis. Endocrinology.

[125] Jiang X.S., Cai M.Y., Li X.J., Zhong Q., Li M.L., Xia Y.F., Shen Q., Du X.G., Gan H. (2022). Activation of the Nrf2/ARE signaling pathway protects against palmitic acid-induced renal tubular epithelial cell injury by ameliorating mitochondrial reactive oxygen species-mediated mitochondrial dysfunction. Front. Med..

[126] Pi J., Leung L., Xue P., Wang W., Hou Y., Liu D., Yehuda-Shnaidman E., Lee C., Lau J., Kurtz T.W. (2010). Deficiency in the nuclear factor E2-related factor-2 transcription factor results in impaired adipogenesis and protects against diet-induced obesity. J. Biol. Chem..

[127] Zhan J., Li X., Luo D., Hou Y., Hou Y., Chen S., Xiao Z., Luan J., Lin D. (2020). Polydatin promotes the neuronal differentiation of bone marrow mesenchymal stem cells in vitro and in vivo: Involvement of Nrf2 signaling pathway. J. Cell. Mol. Med..

[128] Si Z.P., Wang G., Han S.S., Jin Y., Hu Y.X., He M.Y., Brand-Saberi B., Yang X., Liu G.S. (2019). CNTF and Nrf2 Are Coordinately Involved in Regulating Self-Renewal and Differentiation of Neural Stem Cell during Embryonic Development. iScience.

[129] Karkkainen V., Pomeshchik Y., Savchenko E., Dhungana H., Kurronen A., Lehtonen S., Naumenko N., Tavi P., Levonen A.L., Yamamoto M. (2014). Nrf2 regulates neurogenesis and protects neural progenitor cells against Abeta toxicity. Stem Cells.

[130] Xia B., Liu H., Xie J., Wu R., Li Y. (2015). Akt enhances nerve growth factor-induced axon growth via activating the Nrf2/ARE pathway. Int. J. Mol. Med..

[131] Liu J., Wang L., Du Y., Liu S. (2017). An Important Function of Petrosiol E in Inducing the Differentiation of Neuronal Progenitors and in Protecting Them against Oxidative Stress. Adv. Sci..

[132] Pistollato F., Canovas-Jorda D., Zagoura D., Bal-Price A. (2017). Nrf2 pathway activation upon rotenone treatment in human iPSC-derived neural stem cells undergoing differentiation towards neurons and astrocytes. Neurochem. Int..

[133] Sigfridsson E., Marangoni M., Johnson J.A., Hardingham G.E., Fowler J.H., Horsburgh K. (2018). Astrocyte-specific overexpression of Nrf2 protects against optic tract damage and behavioural alterations in a mouse model of cerebral hypoperfusion. Sci. Rep..

[134] Steele M.L., Fuller S., Patel M., Kersaitis C., Ooi L., Munch G. (2013). Effect of Nrf2 activators on release of glutathione, cysteinylglycine and homocysteine by human U373 astroglial cells. Redox Biol..

[135] Kang K.A., Hyun J.W. (2017). Oxidative Stress, Nrf2, and Epigenetic Modification Contribute to Anticancer Drug Resistance. Toxicol. Res..

[136] Kim D.H., Jang J.H., Kwon O.S., Cha H.J., Youn H.J., Chun K.S., Surh Y.J. (2020). Nuclear Factor Erythroid-Derived 2-Like 2-Induced Reductive Stress Favors Self-Renewal of Breast Cancer Stem-Like Cells via the FoxO3a-Bmi-1 Axis. Antioxid. Redox Signal..

[137] Park J., Kim S.K., Hallis S.P., Choi B.H., Kwak M.K. (2021). Role of CD133/NRF2 Axis in the Development of Colon Cancer Stem Cell-Like Properties. Front. Oncol..

[138] Phi L.T.H., Sari I.N., Yang Y.G., Lee S.H., Jun N., Kim K.S., Lee Y.K., Kwon H.Y. (2018). Cancer Stem Cells (CSCs) in Drug Resistance and their Therapeutic Implications in Cancer Treatment. Stem Cells Int..

[139] Lamichhane A., Shahi Thakuri P., Singh S., Rafsanjani Nejad P., Heiss J., Luker G.D., Tavana H. (2022). Therapeutic Targeting of Cancer Stem Cells Prevents Resistance of Colorectal Cancer Cells to MEK Inhibition. ACS Pharmacol. Transl. Sci..

[140] Bai X., Ni J., Beretov J., Graham P., Li Y. (2018). Cancer stem cell in breast cancer therapeutic resistance. Cancer Treat. Rev..

[141] Khan N.M., Ahmad I., Haqqi T.M. (2018). Nrf2/ARE pathway attenuates oxidative and apoptotic response in human osteoarthritis chondrocytes by activating ERK1/2/ELK1-P70S6K-P90RSK signaling axis. Free Radic. Biol. Med..

[142] Sun L., Chiang J.Y., Choi J.Y., Xiong Z.M., Mao X., Collins F.S., Hodes R.J., Cao K. (2019). Transient induction of telomerase expression mediates senescence and reduces tumorigenesis in primary fibroblasts. Proc. Natl. Acad. Sci. USA.

[143] Salminen A., Kauppinen A., Kaarniranta K. (2019). AMPK activation inhibits the functions of myeloid-derived suppressor cells (MDSC): Impact on cancer and aging. J. Mol. Med..

[144] Li R., Liu Y., Shan Y.G., Gao L., Wang F., Qiu C.G. (2019). Bailcalin Protects against Diabetic Cardiomyopathy through Keap1/Nrf2/AMPK-Mediated Antioxidative and Lipid-Lowering Effects. Oxid. Med. Cell. Longev..

[145] Wang Z., Chen Z., Jiang Z., Luo P., Liu L., Huang Y., Wang H., Wang Y., Long L., Tan X. (2019). Cordycepin prevents radiation ulcer by inhibiting cell senescence via NRF2 and AMPK in rodents. Nat. Commun..

[146] Zhao M., Murakami S., Matsumaru D., Kawauchi T., Nabeshima Y.I., Motohashi H. (2022). NRF2 pathway activation attenuates ageing-related renal phenotypes due to alpha-klotho deficiency. J. Biochem..

[147] Wang J., Hu J.Q., Song Y.J., Yin J., Wang Y.Y., Peng B., Zhang B.W., Liu J.M., Dong L., Wang S. (2022). 2′-Fucosyllactose Ameliorates Oxidative Stress Damage in d-Galactose-Induced Aging Mice by Regulating Gut Microbiota and AMPK/SIRT1/FOXO1 Pathway. Foods.

[148] Sun J., Yu X., Huangpu H., Yao F. (2019). Ginsenoside Rb3 protects cardiomyocytes against hypoxia/reoxygenation injury via activating the antioxidation signaling pathway of PERK/Nrf2/HMOX1. Biomed. Pharmacother..

[149] Zheng W., Xie W., Yin D., Luo R., Liu M., Guo F. (2019). ATG5 and ATG7 induced autophagy interplays with UPR via PERK signaling. Cell Commun. Signal..

[150] Chen W., Sun Z., Wang X.J., Jiang T., Huang Z., Fang D., Zhang D.D. (2009). Direct interaction between Nrf2 and p21(Cip1/WAF1) upregulates the Nrf2-mediated antioxidant response. Mol. Cell.

[151] Kang K.A., Piao M.J., Hyun Y.J., Zhen A.X., Cho S.J., Ahn M.J., Yi J.M., Hyun J.W. (2019). Luteolin promotes apoptotic cell death via upregulation of Nrf2 expression by DNA demethylase and the interaction of Nrf2 with p53 in human colon cancer cells. Exp. Mol. Med..

[152] Lapczuk-Romanska J., Wajda A., Pius-Sadowska E., Kurzawski M., Niedzielski A., Machalinski B., Drozdzik M. (2018). Effects of simvastatin on nuclear receptors, drug metabolizing enzymes and transporters expression in Human Umbilical Vein Endothelial Cells. Pharmacol. Rep..

[153] Gao W., Guo L., Yang Y., Wang Y., Xia S., Gong H., Zhang B.K., Yan M. (2021). Dissecting the Crosstalk Between Nrf2 and NF-kappaB Response Pathways in Drug-Induced Toxicity. Front. Cell Dev. Biol..

[154] Dai C., Li B., Zhou Y., Li D., Zhang S., Li H., Xiao X., Tang S. (2016). Curcumin attenuates quinocetone induced apoptosis and inflammation via the opposite modulation of Nrf2/HO-1 and NF-kB pathway in human hepatocyte L02 cells. Food Chem. Toxicol..

[155] El-Shitany N.A., Eid B.G. (2019). Icariin modulates carrageenan-induced acute inflammation through HO-1/Nrf2 and NF-kB signaling pathways. Biomed. Pharmacother..

[156] Shelar S.B., Narasimhan M., Shanmugam G., Litovsky S.H., Gounder S.S., Karan G., Arulvasu C., Kensler T.W., Hoidal J.R., Darley-Usmar V.M. (2016). Disruption of nuclear factor (erythroid-derived-2)-like 2 antioxidant signaling: A mechanism for impaired activation of stem cells and delayed regeneration of skeletal muscle. FASEB J..

[157] Lee Y., Shin J.M., Jang S., Choi D.K., Seo M.S., Kim H.R., Sohn K.C., Im M., Seo Y.J., Lee J.H. (2014). Role of nuclear factor E2-related factor 2 (Nrf2) in epidermal differentiation. Arch. Dermatol. Res..

[158] Kahremany S., Hofmann L., Eretz-Kdosha N., Silberstein E., Gruzman A., Cohen G. (2021). SH-29 and SK-119 Attenuates Air-Pollution Induced Damage by Activating Nrf2 in HaCaT Cells. Int. J. Environ. Res. Public Health.

[159] Helou D.G., Martin S.F., Pallardy M., Chollet-Martin S., Kerdine-Romer S. (2019). Nrf2 Involvement in Chemical-Induced Skin Innate Immunity. Front. Immunol..

[160] D’Autreaux B., Toledano M.B. (2007). ROS as signaling molecules: Mechanisms that generate specificity in ROS homeostasis. Nat. Rev. Mol. Cell. Biol..

[161] Paiva C.N., Bozza M.T. (2014). Are reactive oxygen species always detrimental to pathogens?. Antioxid. Redox Signal..

[162] Vivarini A.C., Lopes U.G. (2019). The Potential Role of Nrf2 Signaling in *Leishmania* Infection Outcomes. Front. Cell. Infect. Microbiol..

[163] Anrather J., Racchumi G., Iadecola C. (2006). NF-kappaB regulates phagocytic NADPH oxidase by inducing the expression of gp91phox. J. Biol. Chem..

[164] Suzuki T., Motohashi H., Yamamoto M. (2013). Toward clinical application of the Keap1-Nrf2 pathway. Trends Pharmacol. Sci..

[165] Kensler T.W., Wakabayashi N., Biswal S. (2007). Cell survival responses to environmental stresses via the Keap1-Nrf2-ARE pathway. Annu. Rev. Pharmacol. Toxicol..

[166] Sykiotis G.P., Bohmann D. (2010). Stress-activated cap’n’collar transcription factors in aging and human disease. Sci. Signal..

[167] Rada P., Rojo A.I., Chowdhry S., McMahon M., Hayes J.D., Cuadrado A. (2011). SCF/beta-TrCP promotes glycogen synthase kinase 3-dependent degradation of the Nrf2 transcription factor in a Keap1-independent manner. Mol. Cell. Biol..

[168] Wu T., Zhao F., Gao B., Tan C., Yagishita N., Nakajima T., Wong P.K., Chapman E., Fang D., Zhang D.D. (2014). Hrd1 suppresses Nrf2-mediated cellular protection during liver cirrhosis. Genes Dev..

[169] Cullinan S.B., Zhang D., Hannink M., Arvisais E., Kaufman R.J., Diehl J.A. (2003). Nrf2 is a direct PERK substrate and effector of PERK-dependent cell survival. Mol. Cell. Biol..

[170] Pekovic-Vaughan V., Gibbs J., Yoshitane H., Yang N., Pathiranage D., Guo B., Sagami A., Taguchi K., Bechtold D., Loudon A. (2014). The circadian clock regulates rhythmic activation of the NRF2/glutathione-mediated antioxidant defense pathway to modulate pulmonary fibrosis. Genes Dev..

[171] Morada M., Pendyala L., Wu G., Merali S., Yarlett N. (2013). Cryptosporidium parvum induces an endoplasmic stress response in the intestinal adenocarcinoma HCT-8 cell line. J. Biol. Chem..

[172] Aldaba-Muruato L.R., Munoz-Ortega M.H., Macias-Perez J.R., Pulido-Ortega J., Martinez-Hernandez S.L., Ventura-Juarez J. (2017). Adrenergic regulation during acute hepatic infection with *Entamoeba histolytica* in the hamster: Involvement of oxidative stress, Nrf2 and NF-KappaB. Parasite.

[173] Ramos S., Carlos A.R., Sundaram B., Jeney V., Ribeiro A., Gozzelino R., Bank C., Gjini E., Braza F., Martins R. (2019). Renal control of disease tolerance to malaria. Proc. Natl. Acad. Sci. USA.

[174] Xu L., Sang R., Yu Y., Li J., Ge B., Zhang X. (2019). The polysaccharide from *Inonotus obliquus* protects mice from *Toxoplasma gondii*-induced liver injury. Int. J. Biol. Macromol..

[175] Vivarini A.C., Calegari-Silva T.C., Saliba A.M., Boaventura V.S., Franca-Costa J., Khouri R., Dierckx T., Dias-Teixeira K.L., Fasel N., Barral A.M.P. (2017). Systems Approach Reveals Nuclear Factor Erythroid 2-Related Factor 2/Protein Kinase R Crosstalk in Human Cutaneous Leishmaniasis. Front. Immunol..

[176] Mukbel R.M., Patten C., Gibson K., Ghosh M., Petersen C., Jones D.E. (2007). Macrophage killing of *Leishmania amazonensis* amastigotes requires both nitric oxide and superoxide. Am. J. Trop. Med. Hyg..

[177] Khouri R., Bafica A., Silva Mda P., Noronha A., Kolb J.P., Wietzerbin J., Barral A., Barral-Netto M., Van Weyenbergh J. (2009). IFN-beta impairs superoxide-dependent parasite killing in human macrophages: Evidence for a deleterious role of SOD1 in cutaneous leishmaniasis. J. Immunol..

[178] Khouri R., Novais F., Santana G., de Oliveira C.I., Vannier dos Santos M.A., Barral A., Barral-Netto M., Van Weyenbergh J. (2010). DETC induces *Leishmania* parasite killing in human in vitro and murine in vivo models: A promising therapeutic alternative in Leishmaniasis. PLoS ONE.

[179] Khouri R., Santos G.S., Soares G., Costa J.M., Barral A., Barral-Netto M., Van Weyenbergh J. (2014). SOD1 plasma level as a biomarker for therapeutic failure in cutaneous leishmaniasis. J. Infect. Dis..

[180] Pourfallah F., Javadian S., Zamani Z., Saghiri R., Sadeghi S., Zarea B., Faiaz S., Mirkhani F., Fatemi N. (2009). Evaluation of serum levels of zinc, copper, iron, and zinc/copper ratio in cutaneous leishmaniasis. Iran J. Arthropod. Borne Dis..

[181] Barreto-de-Souza V., Ferreira P.L., Vivarini Ade C., Calegari-Silva T., Soares D.C., Regis E.G., Pereira R.M., Silva A.M., Saraiva E.M., Lopes U.G. (2015). IL-27 enhances *Leishmania amazonensis* infection via ds-RNA dependent kinase (PKR) and IL-10 signaling. Immunobiology.

[182] Pereira R.M., Teixeira K.L., Barreto-de-Souza V., Calegari-Silva T.C., De-Melo L.D., Soares D.C., Bou-Habib D.C., Silva A.M., Saraiva E.M., Lopes U.G. (2010). Novel role for the double-stranded RNA-activated protein kinase PKR: Modulation of macrophage infection by the protozoan parasite *Leishmania*. FASEB J..

[183] Rath C.T., Schnellrath L.C., Damaso C.R., de Arruda L.B., Vasconcelos P., Gomes C., Laurenti M.D., Calegari Silva T.C., Vivarini A.C., Fasel N. (2019). Amazonian *Phlebovirus* (*Bunyaviridae*) potentiates the infection of *Leishmania* (*Leishmania*) amazonensis: Role of the PKR/IFN1/IL-10 axis. PLoS Negl. Trop. Dis..

[184] Vivarini Ade C., Pereira Rde M., Barreto-de-Souza V., Temerozo J.R., Soares D.C., Saraiva E.M., Saliba A.M., Bou-Habib D.C., Lopes U.G. (2015). HIV-1 Tat protein enhances the intracellular growth of *Leishmania amazonensis* via the ds-RNA induced protein PKR. Sci. Rep..

[185] Vivarini Ade C., Pereira Rde M., Teixeira K.L., Calegari-Silva T.C., Bellio M., Laurenti M.D., Corbett C.E., Gomes C.M., Soares R.P., Silva A.M. (2011). Human cutaneous leishmaniasis: Interferon-dependent expression of double-stranded RNA-dependent protein kinase (PKR) via TLR2. FASEB J..

[186] Lee J., Giordano S., Zhang J. (2012). Autophagy, mitochondria and oxidative stress: Cross-talk and redox signaling. Biochem. J..

[187] Dias B.R.S., de Souza C.S., Almeida N.J., Lima J.G.B., Fukutani K.F., Dos Santos T.B.S., Franca-Cost J., Brodskyn C.I., de Menezes J.P.B., Colombo M.I. (2018). Autophagic Induction Greatly Enhances *Leishmania major* Intracellular Survival Compared to *Leishmania amazonensis* in CBA/j-Infected Macrophages. Front. Microbiol..

[188] Pinheiro R.O., Nunes M.P., Pinheiro C.S., D’Avila H., Bozza P.T., Takiya C.M., Corte-Real S., Freire-de-Lima C.G., DosReis G.A. (2009). Induction of autophagy correlates with increased parasite load of *Leishmania amazonensis* in BALB/c but not C57BL/6 macrophages. Microbes Infect..

[189] Talloczy Z., Jiang W., Virgin H.W.T., Leib D.A., Scheuner D., Kaufman R.J., Eskelinen E.L., Levine B. (2002). Regulation of starvation- and virus-induced autophagy by the eIF2alpha kinase signaling pathway. Proc. Natl. Acad. Sci. USA.

[190] Dias-Teixeira K.L., Calegari-Silva T.C., Medina J.M., Vivarini A.C., Cavalcanti A., Teteo N., Santana A.K.M., Real F., Gomes C.M., Pereira R.M.S. (2017). Emerging Role for the PERK/eIF2alpha/ATF4 in Human Cutaneous Leishmaniasis. Sci. Rep..

[191] Luz N.F., Andrade B.B., Feijo D.F., Araujo-Santos T., Carvalho G.Q., Andrade D., Abanades D.R., Melo E.V., Silva A.M., Brodskyn C.I. (2012). Heme oxygenase-1 promotes the persistence of *Leishmania chagasi* infection. J. Immunol..

[192] Luz N.F., DeSouza-Vieira T., De Castro W., Vivarini A.C., Pereira L., Franca R.R., Silveira-Mattos P.S., Costa D.L., Teixeira C., Meneses C. (2018). *Lutzomyia longipalpis* Saliva Induces Heme Oxygenase-1 Expression at Bite Sites. Front. Immunol..

[193] de Menezes J.P.B., Khouri R., Oliveira C.V.S., Petersen A., de Almeida T.F., Mendes F.R.L., Reboucas A., Lorentz A.L., Luz N.F., Lima J.B. (2019). Proteomic Analysis Reveals a Predominant NFE2L2 (NRF2) Signature in Canonical Pathway and Upstream Regulator Analysis of *Leishmania*-Infected Macrophages. Front. Immunol..

[194] Parmar N., Chandrakar P., Vishwakarma P., Singh K., Mitra K., Kar S. (2018). *Leishmania donovani* Exploits Tollip, a Multitasking Protein, To Impair TLR/IL-1R Signaling for Its Survival in the Host. J. Immunol..

[195] Page A., Volchkova V.A., Reid S.P., Mateo M., Bagnaud-Baule A., Nemirov K., Shurtleff A.C., Lawrence P., Reynard O., Ottmann M. (2014). Marburgvirus hijacks nrf2-dependent pathway by targeting nrf2-negative regulator keap1. Cell Rep..

[196] Edwards M.R., Johnson B., Mire C.E., Xu W., Shabman R.S., Speller L.N., Leung D.W., Geisbert T.W., Amarasinghe G.K., Basler C.F. (2014). The Marburg virus VP24 protein interacts with Keap1 to activate the cytoprotective antioxidant response pathway. Cell Rep..

[197] Kosmider B., Messier E.M., Janssen W.J., Nahreini P., Wang J., Hartshorn K.L., Mason R.J. (2012). Nrf2 protects human alveolar epithelial cells against injury induced by influenza A virus. Respir. Res..

[198] Olagnier D., Peri S., Steel C., van Montfoort N., Chiang C., Beljanski V., Slifker M., He Z., Nichols C.N., Lin R. (2014). Cellular oxidative stress response controls the antiviral and apoptotic programs in dengue virus-infected dendritic cells. PLoS Pathog..

[199] Sun T., Yu H.Y., Zhang C.L., Zhu T.N., Huang S.H. (2018). Respiratory syncytial virus infection up-regulates TLR7 expression by inducing oxidative stress via the Nrf2/ARE pathway in A549 cells. Arch. Virol..

[200] Komaravelli N., Tian B., Ivanciuc T., Mautemps N., Brasier A.R., Garofalo R.P., Casola A. (2015). Respiratory syncytial virus infection down-regulates antioxidant enzyme expression by triggering deacetylation-proteasomal degradation of Nrf2. Free Radic. Biol. Med..

[201] Mastrantonio R., Cervelli M., Pietropaoli S., Mariottini P., Colasanti M., Persichini T. (2016). HIV-Tat Induces the Nrf2/ARE Pathway through NMDA Receptor-Elicited Spermine Oxidase Activation in Human Neuroblastoma Cells. PLoS ONE.

[202] Liu B., Fang M., He Z., Cui D., Jia S., Lin X., Xu X., Zhou T., Liu W. (2015). Hepatitis B virus stimulates G6PD expression through HBx-mediated Nrf2 activation. Cell Death Dis..

[203] Ivanov A.V., Smirnova O.A., Ivanova O.N., Masalova O.V., Kochetkov S.N., Isaguliants M.G. (2011). Hepatitis C virus proteins activate NRF2/ARE pathway by distinct ROS-dependent and independent mechanisms in HUH7 cells. PLoS ONE.

[204] Lee J., Koh K., Kim Y.E., Ahn J.H., Kim S. (2013). Upregulation of Nrf2 expression by human cytomegalovirus infection protects host cells from oxidative stress. J. Gen. Virol..

[205] Choi Y., Jiang Z., Shin W.J., Jung J.U. (2020). Severe Fever with Thrombocytopenia Syndrome Virus NSs Interacts with TRIM21 To Activate the p62-Keap1-Nrf2 Pathway. J. Virol..

[206] Nairz M., Schleicher U., Schroll A., Sonnweber T., Theurl I., Ludwiczek S., Talasz H., Brandacher G., Moser P.L., Muckenthaler M.U. (2013). Nitric oxide-mediated regulation of ferroportin-1 controls macrophage iron homeostasis and immune function in *Salmonella* infection. J. Exp. Med..

[207] Gomez J.C., Dang H., Martin J.R., Doerschuk C.M. (2016). Nrf2 Modulates Host Defense during *Streptococcus pneumoniae* Pneumonia in Mice. J. Immunol..

[208] Joshi C.S., Mora A., Felder P.A., Mysorekar I.U. (2021). NRF2 promotes urothelial cell response to bacterial infection by regulating reactive oxygen species and RAB27B expression. Cell Rep..

[209] Harvey C.J., Thimmulappa R.K., Sethi S., Kong X., Yarmus L., Brown R.H., Feller-Kopman D., Wise R., Biswal S. (2011). Targeting Nrf2 signaling improves bacterial clearance by alveolar macrophages in patients with COPD and in a mouse model. Sci. Transl. Med..

[210] Paiva C.N., Feijo D.F., Dutra F.F., Carneiro V.C., Freitas G.B., Alves L.S., Mesquita J., Fortes G.B., Figueiredo R.T., Souza H.S. (2012). Oxidative stress fuels *Trypanosoma cruzi* infection in mice. J. Clin. Investig..

[211] Pamplona A., Ferreira A., Balla J., Jeney V., Balla G., Epiphanio S., Chora A., Rodrigues C.D., Gregoire I.P., Cunha-Rodrigues M. (2007). Heme oxygenase-1 and carbon monoxide suppress the pathogenesis of experimental cerebral malaria. Nat. Med..

[212] Gramaglia I., Sobolewski P., Meays D., Contreras R., Nolan J.P., Frangos J.A., Intaglietta M., van der Heyde H.C. (2006). Low nitric oxide bioavailability contributes to the genesis of experimental cerebral malaria. Nat. Med..

[213] Ferreira A., Marguti I., Bechmann I., Jeney V., Chora A., Palha N.R., Rebelo S., Henri A., Beuzard Y., Soares M.P. (2011). Sickle hemoglobin confers tolerance to *Plasmodium* infection. Cell.

[214] Ferreira A., Balla J., Jeney V., Balla G., Soares M.P. (2008). A central role for free heme in the pathogenesis of severe malaria: The missing link?. J. Mol. Med..

[215] Cabrales P., Zanini G.M., Meays D., Frangos J.A., Carvalho L.J. (2011). Nitric oxide protection against murine cerebral malaria is associated with improved cerebral microcirculatory physiology. J. Infect. Dis..

[216] Ong P.K., Melchior B., Martins Y.C., Hofer A., Orjuela-Sanchez P., Cabrales P., Zanini G.M., Frangos J.A., Carvalho L.J. (2013). Nitric oxide synthase dysfunction contributes to impaired cerebroarteriolar reactivity in experimental cerebral malaria. PLoS Pathog..

[217] Newton C.R., Warrell D.A. (1998). Neurological manifestations of falciparum malaria. Ann. Neurol..

[218] Jeney V., Ramos S., Bergman M.L., Bechmann I., Tischer J., Ferreira A., Oliveira-Marques V., Janse C.J., Rebelo S., Cardoso S. (2014). Control of disease tolerance to malaria by nitric oxide and carbon monoxide. Cell Rep..

[219] Fourquet S., Guerois R., Biard D., Toledano M.B. (2010). Activation of NRF2 by nitrosative agents and H_2_O_2_ involves KEAP1 disulfide formation. J. Biol. Chem..

[220] Medzhitov R., Schneider D.S., Soares M.P. (2012). Disease tolerance as a defense strategy. Science.

[221] Gozzelino R., Soares M.P. (2014). Coupling heme and iron metabolism via ferritin H chain. Antioxid. Redox Signal..

[222] Pietsch E.C., Chan J.Y., Torti F.M., Torti S.V. (2003). Nrf2 mediates the induction of ferritin H in response to xenobiotics and cancer chemopreventive dithiolethiones. J. Biol. Chem..

[223] Iwasaki K., Mackenzie E.L., Hailemariam K., Sakamoto K., Tsuji Y. (2006). Hemin-mediated regulation of an antioxidant-responsive element of the human ferritin H gene and role of Ref-1 during erythroid differentiation of K562 cells. Mol. Cell. Biol..

[224] Modiano D., Luoni G., Sirima B.S., Simpore J., Verra F., Konate A., Rastrelli E., Olivieri A., Calissano C., Paganotti G.M. (2001). Haemoglobin C protects against clinical *Plasmodium falciparum* malaria. Nature.

[225] May J., Evans J.A., Timmann C., Ehmen C., Busch W., Thye T., Agbenyega T., Horstmann R.D. (2007). Hemoglobin variants and disease manifestations in severe falciparum malaria. JAMA.

[226] Guindo A., Fairhurst R.M., Doumbo O.K., Wellems T.E., Diallo D.A. (2007). X-linked G6PD deficiency protects hemizygous males but not heterozygous females against severe malaria. PLoS Med..

[227] Williams T.N. (2006). Human red blood cell polymorphisms and malaria. Curr. Opin. Microbiol..

[228] Vale P.F., Fenton A., Brown S.P. (2014). Limiting damage during infection: Lessons from infection tolerance for novel therapeutics. PLoS Biol..

[229] Kultz D. (2003). Evolution of the cellular stress proteome: From monophyletic origin to ubiquitous function. J. Exp. Biol..

[230] Kultz D. (2005). Molecular and evolutionary basis of the cellular stress response. Annu. Rev. Physiol..

[231] Lopez-Maury L., Marguerat S., Bahler J. (2008). Tuning gene expression to changing environments: From rapid responses to evolutionary adaptation. Nat. Rev. Genet..

